# Spatial protein expression patterns across pathologically-associated fibers revealed molecular specialization in inclusion body myositis

**DOI:** 10.1186/s12964-026-02720-7

**Published:** 2026-02-21

**Authors:** T. I. Nijssen, S. Davis, R. A. O’Shaughnessy, E. Bos, A. J. van der Kooi, J. Raaphorst, E. Aronica, R. Fischer, B. M. Kessler, Vered Raz

**Affiliations:** 1 Department of Human Genetics, Leiden University Medical Center, Postbus 9600, Leiden, 2300 RC The Netherlands; 2 Centre for Medicines Discovery, Nuffield Department of Medicine, University of Oxford, Oxford, UK; 3 Chinese Academy for Medical Sciences Oxford Institute, Nuffield Department of Medicine, University of Oxford, Oxford, England, UK; 4 Department of Chemical Cell Biology, Leiden University Medical Center, Leiden, The Netherlands; 5 Department of Neurology, Amsterdam University Medical Centre, University of Amsterdam, Amsterdam Neuroscience, Amsterdam, The Netherlands; 6 Department of Neuropathology, Amsterdam University Medical Centre, University of Amsterdam, Amsterdam Neuroscience, Amsterdam, The Netherlands

**Keywords:** Inclusion body myositis, Spatial proteomics, Pathogenesis-associated myofibers, Regenerative myofibers, Histopathology

## Abstract

**Objectives:**

In Inclusion Body Myositis (IBM), myofibers undergo structural and functional changes, including increased regeneration, atrophy, and fibrosis. The molecular mechanisms driving pathologically -associated fibers (PAF) remain poorly understood.

**Methods:**

We developed a myofiber-level proteomic workflow to identify protein signatures of three PAF subtypes. Laser-capture microdissection mass spectrometry of immunolabeled cryosections was performed, complemented by immunofluorescence and electron microscopy validation.

**Results:**

Regenerating fibers expressing embryonic myosin heavy chain showed greater molecular similarity to centrally nucleated fibers than to fibers adjacent to inflammation, which were enriched in aggregation-prone proteins. These distinct proteomic profiles revealed disruptions in protein homeostasis and proteasome composition, implicating impaired proteostasis in defective regeneration. In addition, alterations in HNRNPA1 subcellular localization across PAF subtypes suggest a potential role in driving protein aggregation and inflammation in IBM.

**Conclusions:**

This study underscores the value of spatial proteomics for dissecting localized pathological processes in muscle disease. It highlights the molecular heterogeneity of IBM myofibers and suggests that PAF subtype-specific mechanisms underlie impaired regeneration while pointing to potential drivers of IBM pathology.

**Supplementary Information:**

The online version contains supplementary material available at 10.1186/s12964-026-02720-7.

## Key Message

• This study underscores the value of spatial proteomics for dissecting localized pathological processes in muscle disease.

• It highlights the molecular heterogeneity of IBM myofibers and points to PAF subtype–specific mechanisms underlying impaired regeneration and potential drivers of pathology.

• Regulators of pathology-associated myofibers can be identified as potential targets for translational studies.

**Supplementary Information:**

The online version contains supplementary material available at 10.1186/s12964-026-02720-7.

## Introduction

Skeletal muscle tissue is primarily composed of distinct myofibers that drive contraction and stabilize the skeleton. Myofiber composition adapts in response to exercise, metabolic stress (such as aging), injury, and muscle disease. These adaptations often reflect transitions between fast-twitch glycolytic fibers (expressing MyHC2A, encoded by *MYH2*) and slow-twitch oxidative fibers (expressing MyHC1, encoded by *MYH7*) [1, 2]. In muscle diseases associated with inflammation, histopathological hallmarks include cycles of degeneration and regeneration [3, 4]. Regenerating myofibers are characterized by embryonic MyHC (eMyHC) expression and central nuclei [5, 6]. eMyHC-positive fibers arise from muscle stem cell fusion, whereas centrally nucleated fibers represent an immature state. However, age-related declines in regenerative capacity can impair muscle repair [7]. Understanding the molecular regulators of regenerating myofibers in age-associated muscle diseases could therefore inform targeted therapies.

Inclusion Body Myositis (IBM), the most common immune-mediated muscle disease in adults, is a progressive disorder primarily affecting the finger flexors, quadriceps, and swallowing muscles [8, 9]. IBM pathology is marked by inflammatory infiltrates, rimmed vacuoles, regeneration, and protein aggregates [9–11]. Transcriptomic and proteomic studies have provided insight into mechanisms of myofiber degeneration in IBM [11, 12], and the presence of myogenic factors indicates that regeneration contributes to disease pathology [4]. However, the molecular composition of pathologically-associated fiber (PAF) subtypes in IBM remains poorly defined.

To address this gap, we applied a proteomic strategy focused on three PAF subtypes. Prior studies in IBM and other muscle diseases revealed limited correlation between RNA and protein expression of sarcomeric proteins [13–15], underscoring the need to prioritize spatial proteomics over transcriptomics or bulk proteomics [16, 17]. We therefore used laser capture microdissection mass spectrometry (LCMMS), a powerful approach for defining protein composition in histologically distinct myofibers [16–18]. Notably, LCM-MS has been successfully applied to characterize rimmed vacuoles in IBM [11], allowing direct comparisons.

To elucidate the molecular mechanisms that are associated with PAF in IBM, we collected regenerating fibers based on the histological marks: expression of embryonic MyHC (eMyH-F), and centrally nucleated fibers (CN-F). To study the effect on inflammation on muscle fibers, we collected fibers close to inflammatory regions (Inf-F). We mapped the proteins to each myofiber subtype and = identified PAF-specific protein networks. These results provide new molecular insights into regenerating and inflamed fibers in IBM and may guide the development of future therapeutic strategies.

## Methods

### Human biopsies and cryosectioning

The *vastus lateralis* muscle biopsies were collected from three IBM patients at the Neurology department of Amsterdam University Medical Center (Table S1). The control *vastus lateralis* biopsies were collected at the Radboud Medical Center. Both centres are tertiary referral centres for idiopathic inflammatory myopathies. None of the patients received prior immunosuppressive treatment. The biopsies were immediately fresh-frozen in liquid nitrogen and stored at -80 °C. Biopsies were acquired after written informed consent was given.

Tissues were sectioned to 10 mm using the CM3050-S cryostat (Leica), mounted on Superfrost glass slides (Thermo Fisher Scientific). Cryosections were stored at -20 °C prior to staining.

### Immunohistochemistry

Immunofluorescence was performed as detailed in [19]. Antibodies are listed in Table S2.

#### Laser capture microdissection and proteome analyses

Muscle sections were stained with antibodies to MYH3 and laminin, and DAPI. The laser capture microdissection was performed using a Leica LMD7 in replicates. Fibers were manually selected using the following criteria: in IBM, 1- fibers positive to MYH3, 2- centrally nucleated fibers, and 3- small fibers adjacent to inflammation. In control samples, both large and small muscle fibers were collected. The small fibers were comparable in size to the small fibers located adjacent to inflammatory areas in IBM. For each donor, both fiber subtypes were collected in three replicates, with each replicate defined as a separate sample. Each sample consisted of a pooled laser-capture area of 60,000 μm². This capture area was kept consistent across all samples. The area was consistent across all fiber subtypes. Samples were collected into a tube containing 20 ml RIPA buffer (Thermo Scientific; #89900) for tissue lysis and incubated at room temperature for 60 min. After centrifuging to collect samples in bottom of the tube, a further 20 mL of RIPA containing 25 units of Benzonase (Sigma-Aldrich, E1014-25ku) were added and tubes were centrifuged again to rinse. DTT was added to a final concentration of 5mM, and samples were incubated at room temperature for 30 min. Subsequently, iodoacetamide was added to a concentration of 20 mM and incubated at room temperature for an additional 30 min. Samples were cleaned up and digested using a modified SP3 method, as previously described [20, 21]. Beads (GE45152105050250 & GE65152105050250, Cytiva) were mixed in a 1:1 ratio and 3 ml was added to the RIPA containing protein sample. Acetonitrile was added to a concentration of 70% (v/v), followed by 18 min of incubation at 20 °C while shaking at 1000 RPM. Tubes were placed on magnets for 2 min after which supernatant was discarded. The beads were washed with 70% ethanol (v/v) twice and once with 100% acetonitrile. Next, beads were resuspended in 5 mL 50 mM ammonium bicarbonate containing 5 ng/mL of Trypsin to digest overnight at 37 °C. Samples were mixed to resuspend beads and then placed on the magnet for 5 min. Samples were loaded onto Evotip Pure C18 tips (EvoSep) following the manufacturer’s protocol, and stored at 4 °C until analysis by liquid chromatography mass spectrometry (LCMS/MS).

#### LCMS/MS analysis

Peptides from 40 μm resolution samples were analysed using an Evosep One LC system (EvoSep) coupled to a timsTOF SCP mass spectrometer (Bruker) using the Whisper 40 samples per day method and a 75 μm x 150 mm C18 column with 1.7 μm particles and an integrated Captive Spray Emitter (IonOpticks). Buffer A was 0.1% formic acid in water, Buffer B was 0.1% formic acid in acetonitrile. Data was collected using diaPASEF [21] with 1 MS frame and 9 diaPASEF frames per cycle with an accumulation and ramp time of 100 ms, for a total cycle time of 1.07 s. The diaPASEF frames were separated into 3 ion mobility windows, in total covering the 400–1000 m/z mass range with 25 m/z wide windows between an ion mobility range of 0.64–1.4 Vs/cm2. The collision energy was ramped linearly over the ion mobility range, with 20 eV applied at 0.6 Vs/cm^2^ to 59 eV at 1.6 Vs/cm^2^.

Raw data files were analysed in DIA-NN version 1.8.1 [22] using an in-silico spectral library generated by DIA-NN with default settings using a Uniprot human FASTA file containing 20,383 reviewed sequences, except that a maximum of 2 missed cleavages were allowed. MS1 and MS2 accuracies were set to 15 ppm, all other settings were left as default.

Contamination, including Keratin proteins, was annotated as contam sp and excluded from the raw data. One sample (IBM Inf-F 3.1) had a low number of reads and was excluded for the study. A summary of the data preprocessing and analysis is in figure S1. Preprocessing includes filtering and imputation; both are critical steps in comparative proteomics [23]. Filtering steps: Filter 1 (all samples) – exclusion of counts from fiber subtype replicates when reads were found in only 1 sample, and Filter 2– exclusion of counts from a fiber subtype if reads were found in only one donor. Analysis of IBM signatures was made in Filter 1 dataset, whereas PAF subtype signatures were made in Filter 1 + 2 dataset. Imputation of missing values was made with the impute.QRILC function in the imputeLCMD package, version 2.1 in Rstudio 2024.09.0 build 375 in R version 4.4.0. Quantile regression imputation, QRILC, models the missing values as left-censored and imputes low intensities sampled from the lower tail of an estimated intensity distribution. Intensity distribution showed that after filtering the proportion of detected values increased with protein intensity (Figure S2). QRILC is commonly used in proteomics analysis [24, 25] with similar dataset distribution [26], therefore we considered QRILC an appropriate approach for our data. The imputed data was log2 transformed and normalized by subtracting the median from all measurements.

All subsequent analysis was made in Perseus version 2.0.11. The variation between samples was assessed with principal component analysis (PCA). Differential expression analysis between IBM and control, after filter 1, was done on the average of the three replicates (*N* = 13 samples) with a two-sample t-test with a Benjamini and Yekutieli correction for the false discovery rate (FDR). Differential expression analysis between the three PAF subtypes, after filter 2, was made with a two-way ANOVA corrected for FDR (Benjamini and Yekutieli).

Enrichment analysis of protein networks was conducted in GSEA (4.4.0) and STRING (12.0) using GO: cellular component. The most enriched pathways are reported, considered as most prominent.

#### Literature-based data mining

The published IBM proteomics studies are Parker [27], Li [28], and Gϋttsches [11], and de Vries [29]. Parker, Li and de Vries used bulk protein lysate from muscle biopsy for proteomics. Gϋttsches used LCM-MS on rimmed vacuoles. In all studies, the differential expression analysis was conducted on IBM vs. control samples. In Gϋttsches, Li and Parker studies p-values were not corrected for FDR and a 1.5-fold change cutoff. Gϋttsches reported only the upregulated proteins. The overlap analysis was conducted with 1.5-fold change in de Vries and our study. Proteins that mark vacuoles in IBM were obtained from [30] and [11].

The list of aggregation prone proteins was compiled by combining three studies: one wet lab and two computational predictions. The wet lab proteomic study in C. elegans identified age-associated insoluble proteins [31]. Computational predictions of prion-like proteins used different approaches [32, 33]. In total, the aggregation prone list contained 915 proteins.

For the proteasome complex or ribosome analysis, proteins in the proteome (840 proteins filter-2) were mapped to GO:0000502 (proteasome complex), GO:0005764 (lysosome), GO:0005840 (Ribosome). Differential abundance between fiber subtypes was then assessed using the Benjamini Hochberg (BH) procedure for multiple testing correction. Significant proteasome proteins were subsequently mapped onto a schematic representation of the proteasome structure [34].

#### Imaging and image analysis

After immunostaining the entire muscle cross-section was imaged with the CellInsight CX7 LZR (ThermoFisher) using a 20x objective, or with the Zeiss Axio scan Z1 slide scanner using a 20x objective.

Image analysis was made in Fiji (ImageJ, version 2.15.1).

- For myofiber analysis, fiber region of interest (ROI) was manually selected, and the mean fluorescence intensity (MFI) was measured per channel. Myofibers were selected from comparable areas per sample.
- The MFI and area of immunofluorescence signal were measured after thresholding of the fluorescence signal. Per fluorophore, a constant threshold and comparable areas were used across all samples.
- For the intensity distribution plots, raw images were stacked to RGB, pixels were masked with a Gaussian blur (radius = 1). The intensity plots were made with a line over a region of interest using the *ColorProfiler* plugin in ImageJ.

#### Electron microscopy

Fresh frozen IBM muscle biopsies were thawed on ice for 15 min in Eppendorf tubes. Samples were rinsed with precooled 1 M cacodylate buffer for 5 min and subsequently fixed in 2% paraformaldehyde and 2.5% glutaraldehyde in cacodylate buffer. Biopsies were cut into ~ 2 mm blocks and fixed for 4 h, followed by 1 h post fixation in 1% osmium tetroxide and 1 h incubation in 1% uranyl acetate. Samples were dehydrated through a graded acetone series (70–100%) over 90 min and embedded in Epon. Ultrathin sections (~ 90 nm) were prepared with an ultramicrotome (UC6, Leica, Vienna) using a 35° diamond knife (Diatome, Biel, Switzerland). Sections were collected on formvar- and carbon-coated 1 × 2 mm copper slot grids and stained with 7% uranyl acetate (20 min) followed by lead citrate (10 min). Electron microscopy images were acquired using a Tecnai 12 electron microscope (Thermo Fisher Scientific) equipped with an EAGLE 4k × 4k digital camera. For large area navigation, image montages at 6,500× magnification were generated using stitching software [35].

### Statistical analysis

Statistical analysis and data plotting were made in GraphPad (version 10.2.3) and in R.

## Results

### Establishing a protocol for myofiber-level analysis using LCM-MS

**Fig. 1:**
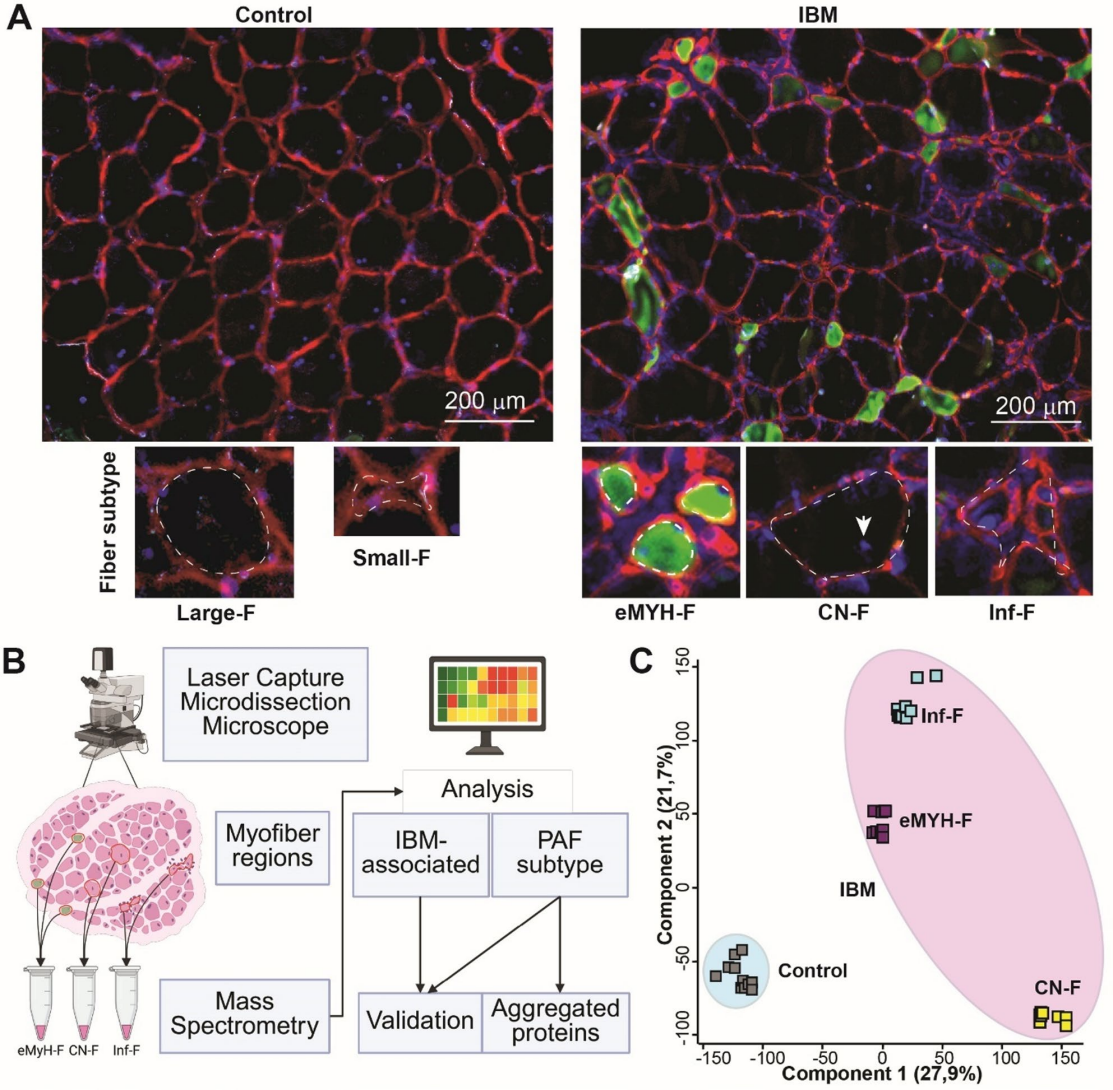
Myofiber-level proteomics in IBM muscles. **A** Representative images from control and IBM muscle cryosections immunostained with laminin (red), eMyHC (green), and nuclei (blue). Scale bar, 200 µm. Lower row: representative images of the five fiber subtypes selected for laser capture microdissection (LCM): in control: large fibers (F) and small fibers (F); in IBM: eMyHC-positive fibers (eMyH-F), centrally nucleated fibers (CN-F), and inflammation-adjacent fibers (Inf-F). Dashed lines indicate capture area examples; arrowhead marks a central nucleus. **B** A flowchart summarizes the main steps in the myofiber-level study to define signatures associated with IBM and pathologically associated fibers (PAFs). **C** Principal component analysis (PCA) of variations among 38 samples in control and IBM samples in the Filter 1 dataset. Control samples are circled in blue; IBM samples in pink

To identify the proteome of pathogenic fiber subtypes in IBM, three fibre subtypes were collected: eMyHC-positive and centrally nucleated fibers, which represent two stages of regeneration, and small fibers in inflammatory regions, which represent an inflamed or atrophying state (Fig. 1A). Healthy, mature myofibers were collected from healthy donors. Both large and small fibers were collected: the small fibers were similar in size to those in inflammatory-rich regions (Fig. 1A). In IBM, we collected central nucleated fibers (CN-F), which were similar in size to the large fibers in the control group. We also collected two smaller fiber subtypes: fibers positive for eMyHC (eMyHC-F) and a subtype adjacent to inflammatory infiltrates (Inf-F), which were negative for eMyHC (Fig. 1A). Fiber subtypes were manually captured. The same capture area was collected for each sample, with each sample containing multiple laser-captured area (Fig. 1B). Three samples were collected per donor, and a total of 39 samples were subjected to mass spectrometry. One sample was excluded due to low read counts. Preprocessing included filtering and imputation. To reduce potential noise, counts detected in only one replicate from the same donor were excluded (Filter 1). We selected the imputation protocol based on the distribution of valid values in the dataset. Comparison of intensity distribution plots between the raw and filtered data showed that, after filtering, the proportion of detected values increased with increasing protein intensity (Figure S2), making the data suitable for QRILC imputation [36]. Subsequently, the data were normalized, and all analyses were performed on the averaged values across replicates.

Major variances between the samples (*N* = 38) was assessed with the principal component analysis. A clear separation between IBM and control samples was found with component 1 (27.9% variance; Fig. 1C). Component 2 showed small variance between control and CN-F but high variance between CN-F and eMyHC and Inf-F. PCA revealed clustering of the three PAF subtypes in IBM (Fig. 1C). The large and small control myofibers were undistinguishable (Fig. 1C), further differential expression analysis showed no significant differences (Figure S3). Therefore, the two control myofiber groups were combined for downstream analyses.

### Myofiber-level analysis revealed reduced muscle function

**Fig. 2:**
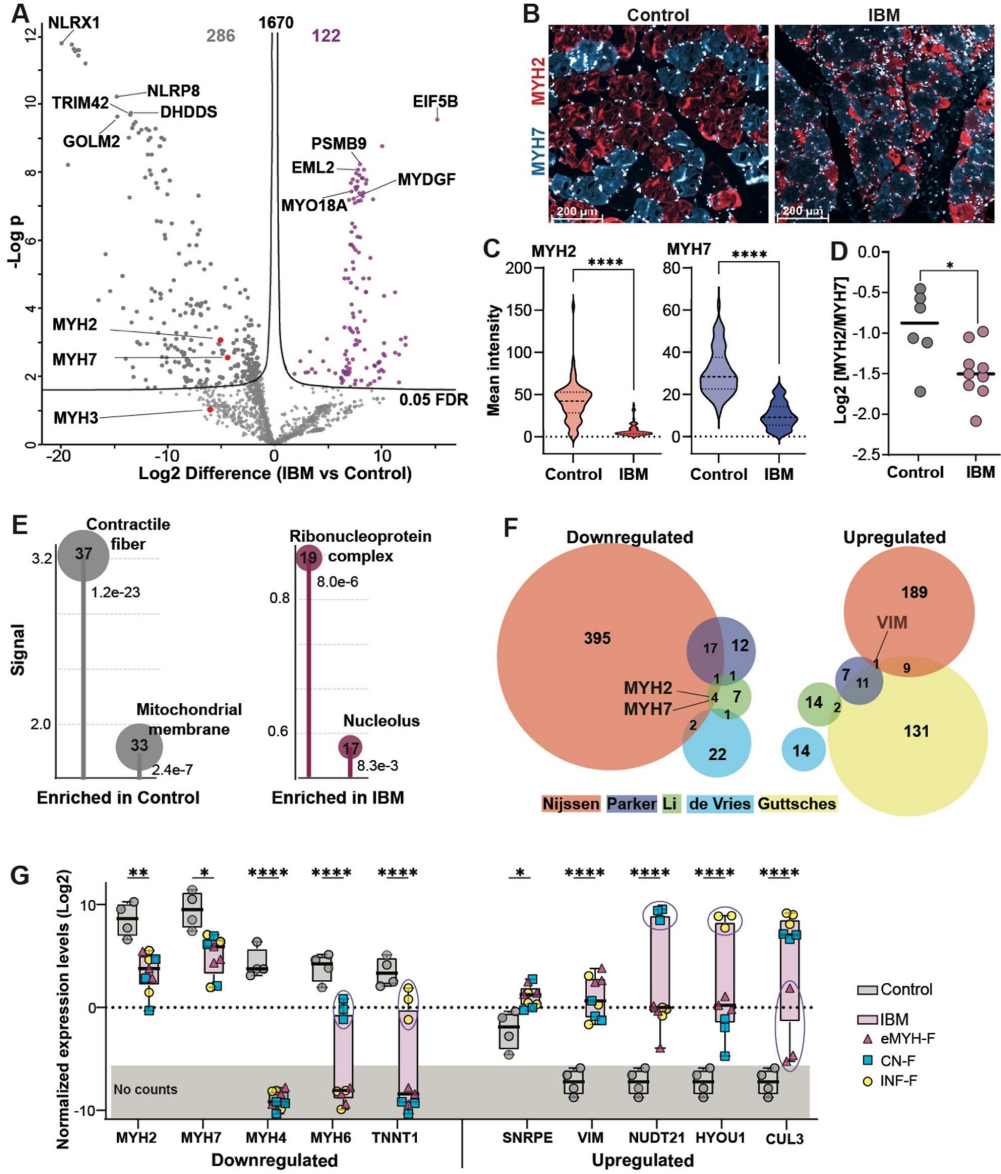
IBM protein signatures. **A** Volcano plot showing differential protein expression between IBM and control muscles. The black line indicates the 5% false discovery rate (FDR) threshold. Numbers of differentially expressed proteins are indicated in each quadrant. Proteins uniquely expressed in control or IBM are shown as grey and pink circles, respectively. The top 5 DE proteins are depicted. **B**-**C** Representative immunofluorescence images and violin plots of MYH2 (red) and MYH7 (blue) fluorescence intensity (MFI) in control and IBM muscles. Nuclei are counterstained in white. Scale bar, 200 µm. **D** Dot plot of the ratio between MYH2 and MYH7, log2 expression values in control and IBM samples. **E** Lollipop plots of the most enriched protein networks among downregulated and upregulated proteins. The y-axis shows the calculated Signal (in STRING) of GO: Cellular Component. indicated. The bubble size corresponds to protein count in each network and the p-value FDR is denoted under the bubble. **F** Venn diagram showing overlap of IBM protein signatures between this study (Nijssen) and published IBM proteomics studies (Li, Parker, de Vries, Güttsches). **G** Dot plots of normalized expression levels for selected down- and upregulated proteins. Encircled dots indicate samples from the same PAF subtype. Gray shading marks areas with no reads. Statistical significance (one-way ANOVA, FDR-adjusted): *p* < 0.05 (*), < 0.01 (**), < 0.00001 (***)

Data analysis was made with the average of the triplicate samples: IBM (*N* = 9) and control (*N* = 4). Differential expression analysis identified 408 proteins with significant changes (*p* < 0.05, FDR), 286 proteins (70%) were downregulated and 30% were upregulated in IBM (Fig. 2A). Among the top downregulated proteins, both NLRX1 and NLRP8 trigger a protective inflammatory response [37, 38]. Among the top five upregulated proteins, EIF5B is implicated as a driver of stress-related translation of the immune response [39]. and upregulation of PSMB9, an immune-associated catalytic proteasomal subunit, is associated with several immune diseases [40]. Downregulation of MYH7 and MYH2 was validated by immunofluorescence (Figs. 2B–C, Figure S4). A switch between fast- and slow- twitch myofibers, is mechanical indicator of myofiber vulnerability in disease condition [41] and can be calculated by the ratio between MYH2 and MYH7. The ratio MYH2 / MYH7 was significantly reduced in IBM (Fig. 2D), in agreement with muscle weakness. In contrast, MYH3 (eMYHC), although expressed in a subset of IBM fibers, was not significantly altered at the proteomic level (Fig. 2A).

To interpret the biological significance of the IBM-associated proteome, we investigated the enrichment of IBM-dysregulated proteins or the ranking of IBM-associated proteins within protein networks using STRING or GSEA, respectively. Both analyses revealed significant depletion of the sarcomere and mitochondrial networks. This is consistent with the reduced expression of the structural and metabolic machinery underlying muscle weakness (Fig. 2E, Table S3, and Figure S5). IBM-upregulated proteins were enriched for RNA-related processes, including RNA-binding proteins, ribonucleoproteins, and nucleolar functions (Fig. 2E and Table S3). However, the endocytic vesicles and nuclear protein networks were identified using GSEA (Figure S5). These networks included both up- and down-regulated proteins (Figure S5); therefore, they were not detected by STRING analysis. The enrichment of endocytic vesicles aligns with the vacuole pathology characteristic of IBM. The absence of immune-related protein networks suggests that immune cells were not, or only sporadically, captured by laser.

To relate our study to published proteomic studies in IBM, we examined the overlap of up or down regulated proteins between our study and four IBM proteomics studies, Li, Güttsches, Parker and deVries [11, 27–29]. Only limited protein overlap was found across all five studies (Fig. 2F). The downregulated proteins that overlapped were predominantly sarcomeric, including MYH2 and MYH7 validated proteins (Table S4). There was minor overlap between the upregulated proteins, and vimentin (VIM), a structural protein involved in cell mechanics, was also found in the Güttsches and Parker studies (Fig. 2F). The limited overlap between the five studies may be due to differences in study design, laboratory procedures, and technical methods. For example, Li, Parker, and DeVries extracted proteins from entire muscle biopsies, whereas Güttsches and we focused on laser-captured fibers of different subtypes. Proteomics outcomes are greatly affected by sample processing and the depth of data acquisition. Furthermore, even large-scale proteomic technologies are not genome-wide in coverage, and comparative studies are biased by specific mass spectrometry protocols, which limits the number of disease-associated overlapping proteins [42].

To verify the differential expression analysis, we plotted the normalized expression values of several down- or upregulated proteins, as examples. Some the downregulated proteins, such as MYH2 and MYH7, exhibited lower expression levels in IBM samples, whereas others were not expressed in all IBM samples, such as MYH4, or absent from a subset of IBM samples, such as MYH6 and TNNT1 (Fig. 2G). Among the upregulated proteins, SNRPE showed higher expression levels in IBM across all samples. In contrast, the expression of other upregulated proteins was driven by a lack of expression in the control group (Fig. 2G). For instance, VIM, which is upregulated in three IBM studies, is expressed in all IBM samples, regardless of PAF subtype. However, NUDT21, HYOU1, and CUL3 were expressed at higher levels across all IBM samples, though expression differed between fiber subtypes (Fig. 2G). These results suggest differences in protein composition among IBM-PAF subtypes.

### Differences in protein composition distinguish between pathologically affected fibers

To investigate molecular differences between PAF subtypes and identify PAF-specific signatures, we added Filter 2, which discards counts from a fiber subtype if reads are present in only one patient. After applying Filter 2, 807 proteins remained for analysis (Figure S1). To assess the effect of Filter 2, we compared principal component analysis (PCA) and differential expression analysis between Filter 1 and Filter 1 + 2 (Figure S6A–B). PCA of the Filter 1 and Filter 1 + 2 datasets showed comparable clustering of IBM and control samples and PAF subtypes (Figure S6A–B). Notably, a volcano plot of the differential expression analysis using the Filter-1 + 2 dataset revealed distinct protein clusters (“wings”) composed of proteins expressed in one, two, or all three PAF subtypes. These clusters were not observed with Filter-1 (Figure S6). MYH3 was identified in a protein cluster expressed in a single PAF subtype (Figure S7B). Col6A1 emerged as a significant protein with Filter 1 + 2 but not with Filter 1 (Figure S6A–B). Immunofluorescence of collagen confirmed higher levels in IBM compared with the control group (Figure S6C), indicating that Filter 2 is more reliable for identifying proteins that are differentially expressed between PAF subtypes.

**Fig. 3:**
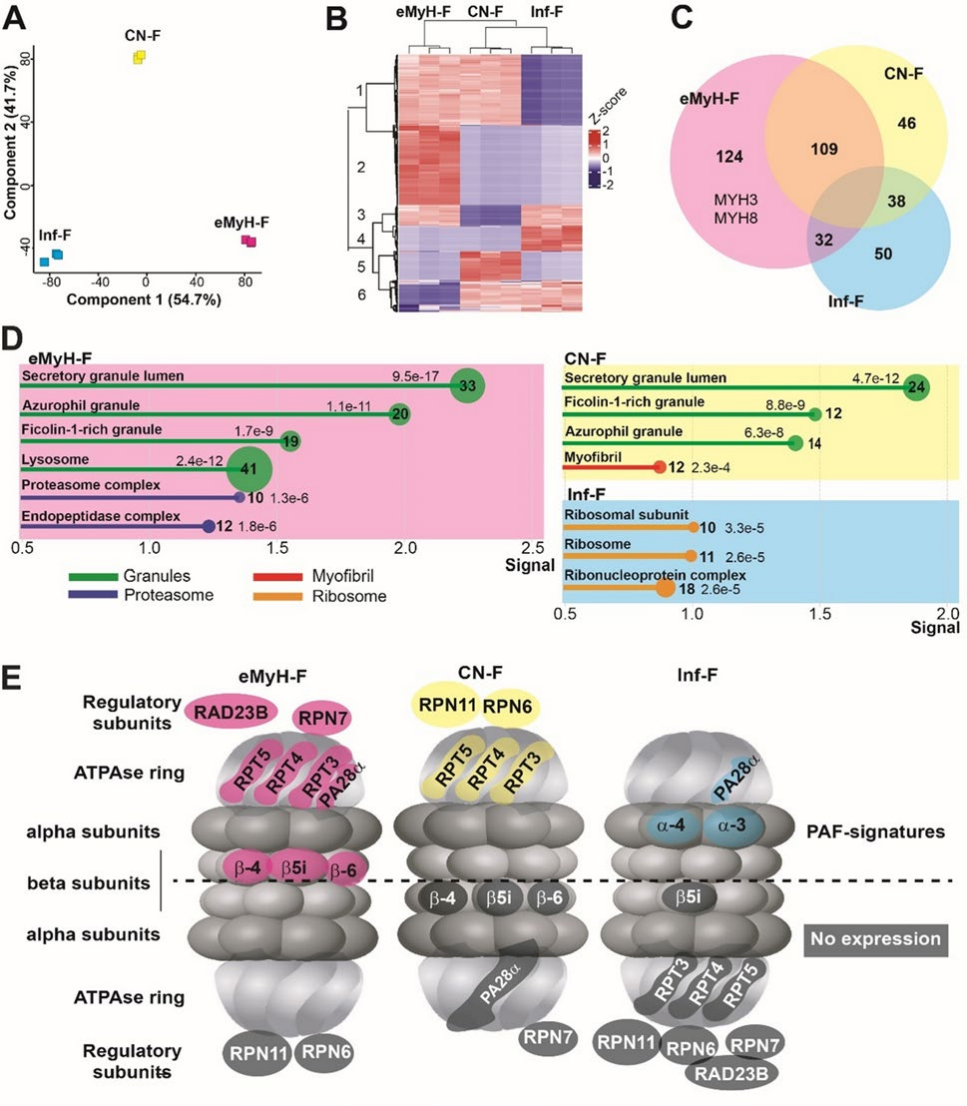
Protein signatures in IBM pathogenesis-associated fibers (PAF). Color code: CN-F (yellow), eMyH-F (magenta), Inf-F (cyan). **A** Principal component analysis (PCA) of PAF subtypes. **B** A Heatmap of z-score of protein signatures (*p* < 0.05, FDR corrected) in eMyH-F, CN-F and Inf-F. Rows represent proteins and columns represent samples. Clustering based on samples shows higher similarity of expression levels between CN-F and Inf-F. Clustering based on protein levels shows six groups: 1-, 4-, and 5- proteins expressed in eMyH-F, Inf-F or CN-F, respectively; 2-, 3-, and 6- proteins expressed in eMyH-F&CN-F, eMyH-F & Inf-F, or CN-F and Inf-F, respectively. **C** Venn diagram shows the number of overlapping and unique of protein signatures in eMyH-F, CN-F and Inf-F. **D** Lollipop plots of enriched GO:cellular component pathways. X-axis shows signal value from String; for each pathway, the number of associated proteins and adjusted *p*-values (FDR) are indicated. Pathways are color-coded: cellular granules (green), myofibril (red), proteasome (dark blue), ribosome (orange). **E** Scheme of the proteasome complex with dysregulated proteins highlighted in IBM PAF subtypes. PAF-unique proteins are colored; proteins not detected are shown in black and white

PCA of the three fiber subtypes distinguished the PAF subtypes (Fig. 3A). We used a two-way ANOVA to identify fiber subtype signatures. A heatmap of their expression levels revealed six groups, including proteins unique to each fiber subtype and proteins expressed in two fiber subtypes (Fig. 3B; see Table S5 for a list of the unique signatures). MyH-F exhibited the greatest number of protein signatures (265 proteins). Notably, the developmental myosins MYH3 and MYH8 were significantly enriched in MyH-F, which supports the analysis pipeline (see Figure S6). In contrast, CN-F and Inf-F had only 193 and 120 signatures, respectively (Fig. 3C; Table S5). MyH-F signatures were specifically enriched for lysosome, proteasome, and endopeptidase protein networks, whereas CN signatures were specifically enriched for muscle proteins (Fig. 3D and Table S6). These results suggest that eMyH-F has higher control of protein homeostasis, while CN-F has higher myofiber maturation. One hundred and nine proteins overlapped between eMyH-F and CN-F and were enriched in granule protein networks (Fig. 3D). Inf-F signatures showed the least protein overlap (Fig. 3C), and their signatures were enriched in ribosome and ribonucleoprotein networks (Fig. 3D). This suggests that the regulation of protein homeostasis might differ between eMyH-F and Inf-F.

To evaluate the impact of proteasome expression patterns on activity in each PAF subtype, we mapped proteasome-associated signatures onto proteasome subunits. The β-subunit, which is involved in catalytic activity, was highly expressed in eMyH-F, but its expression was lacking in CN-F and InF. Moreover, PA28α expression was depleted in CN-F (Fig. 3E). The ATPase ring proteins and three deubiquitinating enzymes were absent from InF-F, while different regulatory subunits were enriched in eMyH-F and CN-F (Fig. 3E). Most strikingly, the immunoproteasome subunit β5i was exclusively detected in eMyH-F. Together, these findings suggest that proteasome activity is differentially regulated across PAF subtypes and may be impaired in CN-F and Inf-F.

Since vacuole protein networks are a histopathological feature of IBM and are enriched in both eMyH-C and CN-F, we examined whether known vacuole protein markers are enriched in each fiber subtype. However, none of the validated vacuole markers identified by [30] were found in our study. Four vacuole proteins identified in the Guttsches study were found to be eMyH-F signatures. However, none were found in CN-F or Inf-F (Table S4), which suggests that we did not select vacuolated myofibers in our study.

### Aggregation-prone proteins are differentially expressed in PAF subtypes

**Fig. 4:**
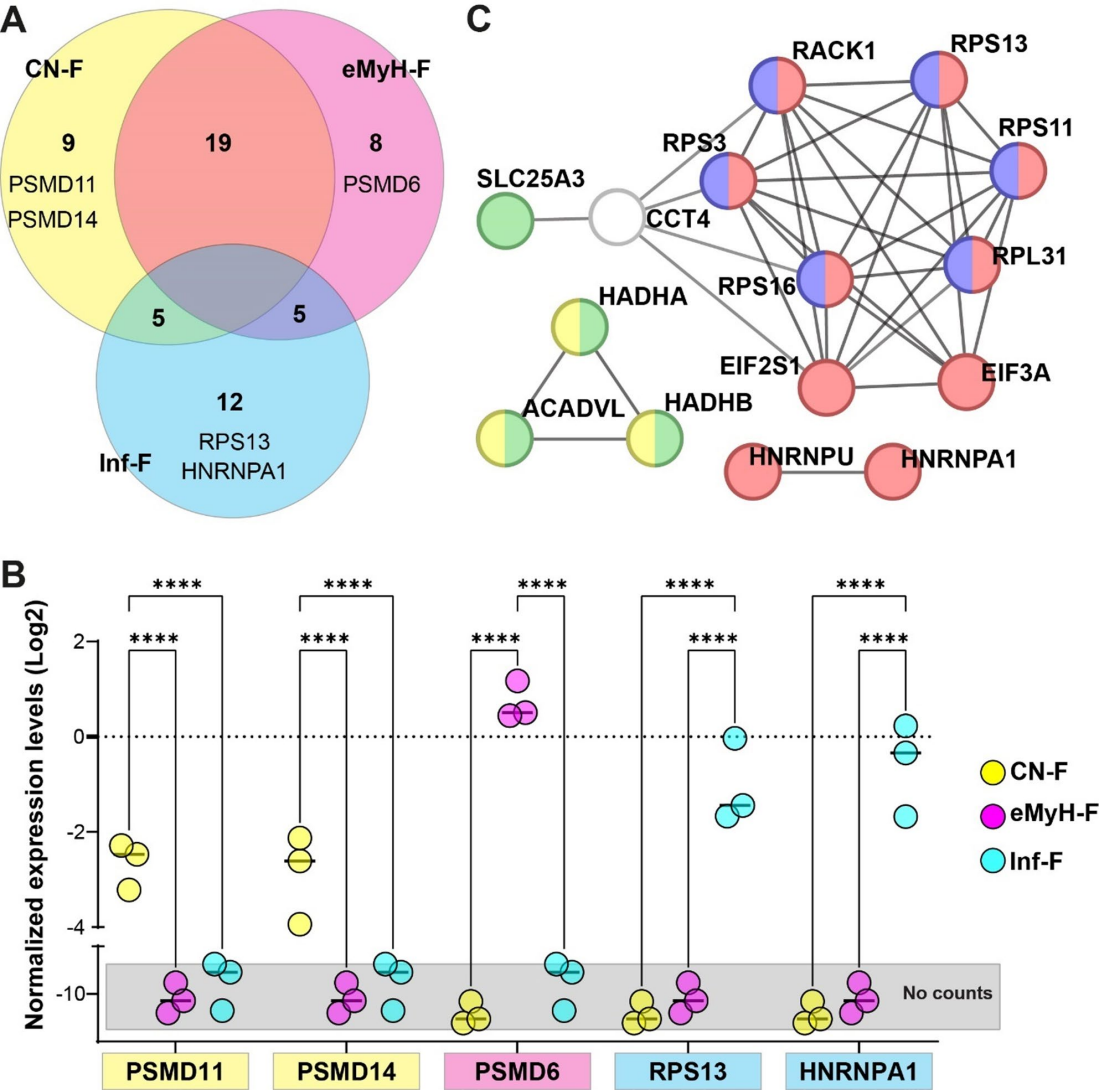
Aggregation-prone proteins in Pathogenesis-Associated Fibers (PAF). **A** Venn diagram of aggregation-prone protein signatures. **B** Dot plots of normalized expression levels for selected PAF-unique proteins. Gray shading marks areas with no reads. Statistical significance (one-way ANOVA, FDR-adjusted): **** *p* < 0.00001. **C** Protein-protein interaction (PPI) network of aggregation-prone protein signatures from Inf-F. Functional annotations: ribonucleoprotein complex (red), ribosome (blue), mitochondrial inner membrane (green), and β-oxidation of palmitoyl-CoA to myristoyl-CoA (yellow)

Protein aggregation is a molecular hallmark of IBM, but aggregation-prone proteins have not been systematically compared across fiber subtypes. To address this, we compiled a literature-based list of aggregation-prone proteins and examined their distribution across PAF (Table S7). Nineteen proteins were shared between CN-F and eMyH-F, underscoring their molecular similarity, while only ~ 30% were unique to each subtype (Fig. 4A). In contrast, Inf-F displayed the most distinct profile, with 55% of proteins unique to this subtype, including HNRNPA1 (Fig. 4A). Expression plots for selected proteins confirmed PAF-specific patterns (Fig. 4B).

To further characterize aggregation-prone proteins in Inf-F, we constructed a protein–protein interaction (PPI) network, revealing three major clusters: mitochondrial inner membrane proteins, including a subgroup involved in β-oxidation of fatty acids, including conversion of palmitoyl-CoA to myristoyl-CoA; ribosomal proteins; and heterogeneous nuclear ribonucleoproteins, including HNRNPA1 and HNRNPU (Fig. 4C). Collectively, these findings suggest that aggregation-prone proteins disrupt distinct cellular machineries in a fiber subtype–specific manner.

### Molecular specialization across pathology-associated fiber subtypes was confirmed by immunofluorescence and electron microscopy

**Fig. 5:**
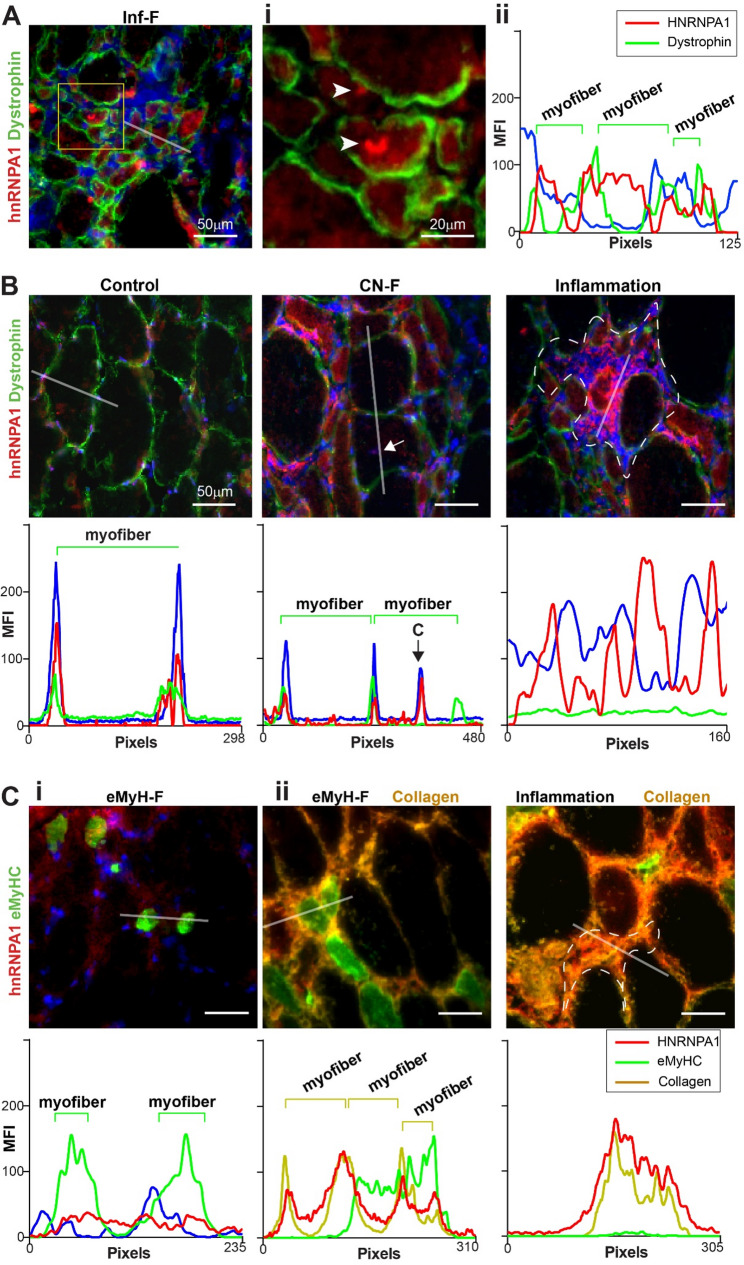
Validation of elevated HNRNPA1 signal in Inf-F with extracellular localization in IBM muscle. Representative images are from P2. **A**-**B** HNRNPA1 (red) and dystrophin (green) immunofluorescence. nuclei are blue. **A** Inf-F: the region selected for analysis is indicated by a white line. Yellow box highlights HNRNPA1 aggregation near inflammatory regions; zoom shown in (i). MFI profile (ii). **B** Control myofibers, centrally nucleated fibers (CN-F), and inflammation-adjacent regions in IBM. Green line marks dystrophin-associated myofiber boundary through which the analysis line passes. White arrow marks a central nucleus; dashed white line outlines inflammatory region. Scale bar, 50μm. **C** HNRNPA1 (red) and eMyHC (green) immunofluorescence in eMyHC-positive fibers (eMyH-F) and inflammation-adjacent regions. Collagen (yellow) labels extracellular matrix (ii). White lines indicate regions selected for MFI analysis; corresponding plots shown below each image. In MFI plots, green (dystrophin) and yellow (collagen) lines mark fiber boundaries crossed by the analysis line. Nuclei are blue. Scale bar, 50μm

Among aggregation-prone proteins, HNRNPA1 is notable for its intrinsic prion-like properties, which drive cytoplasmic mislocalization and the formation of toxic amyloid fibrils implicated in neurodegenerative diseases such as amyotrophic lateral sclerosis (ALS) and multisystem proteinopathy [43]. HNRNPA1 immunohistochemistry in IBM showed higher signal in Inf-F, with fluorescent puncta suggestive of aggregates (Fig. 5A, and Figure S7). In Inf-F, HNRNPA1 was localized to the cytoplasm, whereas in control muscles and CN-F the signal was predominantly nuclear (Fig. 5B). Notably, in inflamed regions nuclear localization was lost altogether (Fig. 5B). Consistent with LCMMS results, HNRNPA1 levels were low in eMyH-F (Fig. 5Ci). However, colocalization with collagen suggested an extracellular localization in these fibers (Fig. 5Cii). The spatial relocalization of HNRNPA1 was consistent across all three IBM patients (Figure S8).

**Fig. 6:**
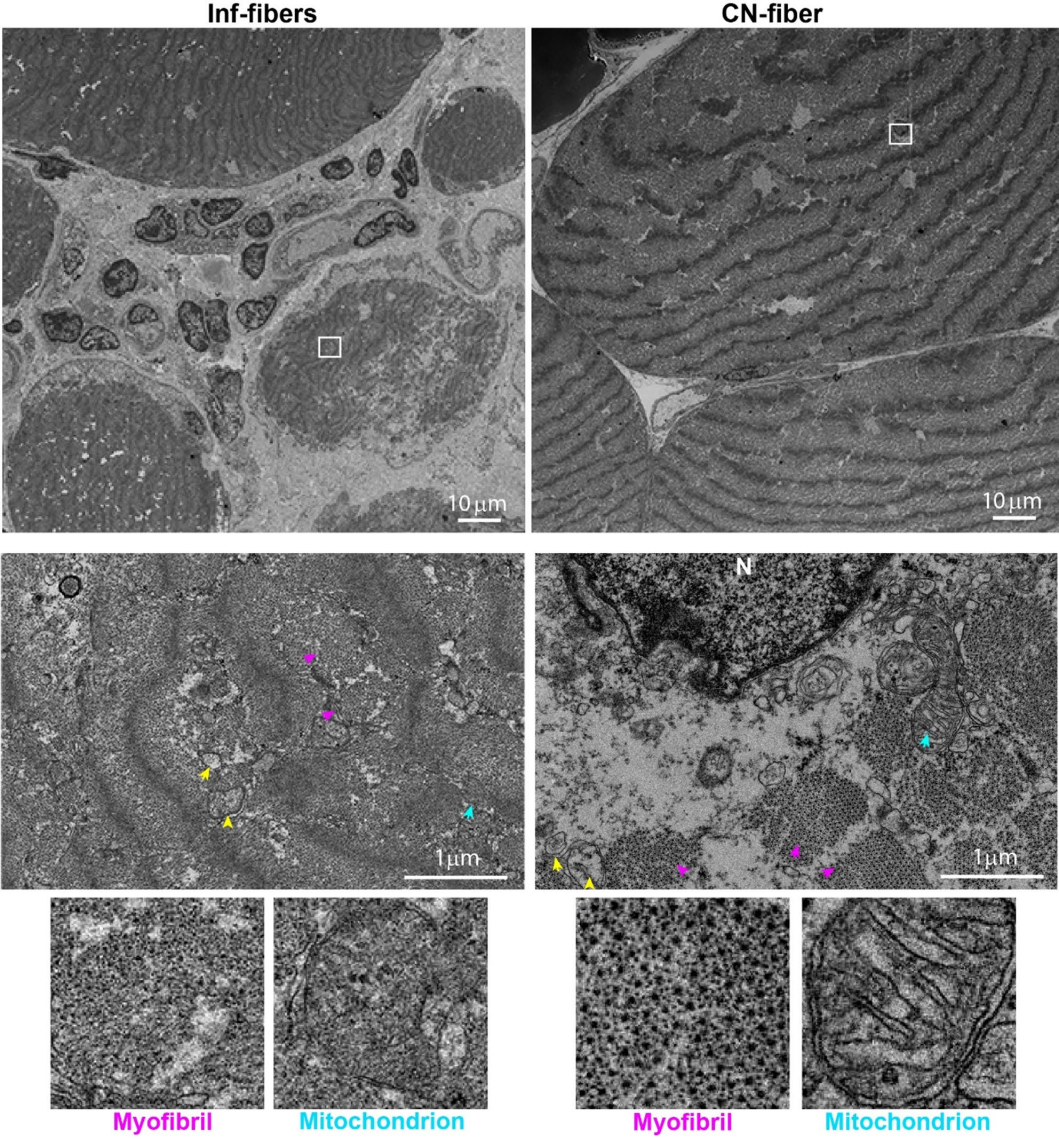
Distorted myofibril and mitochondrial structures in Inf-F, but not in CN-F. Cross sectional view of electron microscopy from IBM VL muscle. Representative images of centrally nucleated (CN) fibers and inflammation-adjacent (Inf) fibers. Scale bar, 10µm. Insets show selected regions at 10× magnification (middle row, scale bar 1 µm). Yellow arrowheads indicate vacuoles; purple arrows indicate myofibrils; cyan arrows indicate mitochondria. Bottom row: high-magnification images of a single mitochondrion (showing cristae) and a single myofibril (showing myosin and actin filaments)

The differential enrichment of myofibril proteins in CN-F intrigued us to examine myosin and actin filaments architecture using electron microscopy (EM). Fresh-frozen muscle biopsies showed only minor freezing artifacts in stitched cross-sectional overviews (Figure S9). Centrally nucleated fibers displayed a well-organized ultrastructure of myosin and actin filaments, characterized by a repeated pattern (Fig. 6). This pattern is essential for contraction [44]. In contrast, in Inf-F the actin and myosin filaments were disordered and lacked the regular pattern (Fig. 6). In addition, we noticed that the mitochondria in CN-F showed intact cristae, consistent with preserved function, but in Inf-F mitochondria appeared structurally compromised, with loss of visible cristae (Fig. 6). Consistent with proteomic findings, intracellular vacuoles were observed in CN-F and Inf-F (Fig. 6). The EM study highlights structural differences between CN-F and Inf-F.

## Discussion

In this study, we used spatial proteomics with LCMMS to characterize the protein composition of PAF subtypes in IBM. We discriminated between PAF-unique signatures and overlapping proteins between two PAF subtypes. The eMyH- and CN- fibers shared a higher degree of molecular similarity than Inf-F, which is consistent with their representation of two stages in myofiber regeneration [45]. The overlapping proteins of eMyH-F and CN-F were mapped to vacuole and granule networks, suggesting that these protein networks play a role in regeneration. The expression of developmental MyHCs (MYH3 and MYH8) in eMyH-F, but not in CN-F, indicates that these fibers represent a different stage of regeneration compared with CN-F. Moreover, protein networks involved in the regulation of protein homeostasis were specifically enriched in eMyH-F, suggesting a role for these pathways in early muscle regeneration. Consistent with this, proteasomal, lysosomal, and vacuole proteins have been reported as markers of cellular remodelling and stress response [46]. However, as expected in myofibers undergoing maturation, CN-F signatures were enriched for myofibril-associated proteins. Electron microscopy confirmed the presence of structured sarcomeres in the CN-F. In contrast, Inf-F showed elevated levels of RNA-binding and ribosomal proteins, as well as different proteasome composition, suggesting different regulation of protein homeostasis in Inf-F and eMyH-F. Most notably, PSMB8, an immune proteasome protein that induces proteasome malfunction and neurotoxicity [47], was highly expressed specifically in eMyH-F. Both eMyH-F and Inf-F were found in areas rich in inflammation, but the presence of PSMB8 only in eMyH-F suggests that Inf-F is less immune responsive than eMyH-F. The small size of Inf-F could suggest atrophy. Reduced expression of sarcomeric and mitochondrial proteins, together with enrichment of ribosomal proteins, has been reported in muscle atrophy [14]. The differential expression of proteins in cellular machinery regulating proteostasis, ribosome, lysosome and proteasome across the three PAF, suggests myofiber specificity.

In addition to myofiber regeneration and inflammation, IBM is characterized by rimmed vacuoles containing aggregated β-amyloid [48]. Although we did not find rimmed vacuoles in our IBM proteome, we did find an enrichment of aggregation-prone proteins, specifically in the Inf-F subtype. Protein aggregation is a hallmark of IBM. Notably, RNA-binding proteins make up just 1% of the human proteome but represent approximately 30% of aggregation-prone proteins [49]. In our study, HNRNPA1 was one of the most highly expressed aggregation-prone RNA-binding proteins. We observed relocalization of HNRNPA1 between CN-F and Inf-F. HNRNPA1 was found to be nuclear localized in healthy individuals, whereas in Inf-F and inflamed regions, it was found to be punctate, nuclear depleted, and redistributed to the extracellular space, overlapping with collagen. Recent work has linked cell-surface RNA-binding proteins, such as HNRNPA1, to immune activation [50]. This suggests that mislocalization of HNRNPA1 contributes to immune recruitment in IBM. Nuclear mislocalization of HNRNPA1 aligns with its involvement in stress granule dynamics and aggregation [51]. Disturbances in stress granules dynamics have been implicated in IBM and other neuromuscular protein aggregation diseases [52]. The association between HNRNPA aggregation and cytoplasmic accumulation suggests its malfunction in RNA processing, particularly in neurodegenerative diseases [53, 54]. The nuclear localization sequence in HNRNPA1 forms fibril structures, suggesting a functional role in pathological aggregates [43]. Our results support a role for HNRNPA in IBM disease mechanisms.

Proteomics datasets are strongly shaped by study design, technical variation, and sample handling, and experiments typically contain missing values [55, 56]. These factors likely contribute to the limited overlap in protein composition reported across four IBM proteomic studies, which did not yield a unified IBM signature. Still, all studies, including ours, consistently showed a reduction in sarcomere proteins, despite the small number of overlapping muscle proteins. We acknowledge that the modest sample size in our IBM versus control analysis may constrain the generalizability of IBM-specific protein signatures. Computational approaches such as filtering and imputation are necessary to address mass spectrometry limitations, though they can also introduce bias. Here, we applied two filtering strategies to reduce low-confidence proteins. Filter-1 preserved a normal distribution across samples, while Filter-2 uncovered protein clusters indicative of discontinuity in IBM datasets, consistent with the presence of three PAF subtypes. Filter-2 enabled identification of discrete clusters containing proteins expressed in one, two, or all PAF supported by immunohistochemistry and electron microscopy validation. Missing values, often reflecting low-abundance peptides falling below the detection limit of mass spectrometry and experimental variation, reduce statistical power and limit reproducibility. Therefore, dataset imputation is a common practice in proteomic analysis [57]. In general, single-imputation methods, including QRILC, can shrink or distort differences between conditions for proteins with many missing values especially when the patter of missingness differs between those conditions [25]. Several imputation protocols are available for mass-spectrometry data [57], and the intensity distribution can inform the choice of the most appropriate method. In our dataset, the proportion of detected values increased with protein intensity, rendering the data suitable for QRILC imputation. This enables the inclusion of features with incomplete data while introducing systemic bias by reducing variance and inflating potential differences among low-intensity features. A high proportion of imputed values should be interpreted with caution. To reduce the proportion of imputed data, we applied filters and used the average of replicates for analysis. Our validation of collagen enrichment in IBM in filter 2 and the myofiber subtype-specific expression of MYH3 and MYH8 suggest that our data preprocessing protocol was appropriate. At the pathway level, enrichment analyses are likely to be driven by the well-observed proteins across the majority of samples and should be relatively robust to the imputation method. However, pathways supported mostly by low-abundance and heavily missing proteins should be interpreted with caution. For this reason, we reported only the most prominent enriched pathways.

Our study demonstrates the molecular heterogeneity of pathogenic myofibers in IBM and identifies potential protein markers for future translational research. Distinct profiles of regenerating fibers and inflamed regions suggest that impaired protein homeostasis contributes to defective regeneration. In this study, however, we focused on only three PAFs in IBM. Future studies should explore a wider range of pathologically associated myofibers, as well as the proteomics of healthy myofibers under disease conditions. In addition, this study did not investigate the molecular differences between pathologically affected myofibers and intact or healthy myofibers. Such analyses would require inclusion of intact myofibers that express the mature myosins (Type I, IIA, II) from the same muscle biopsy. Together, these findings underscore the value of spatial proteomics in revealing localized pathological mechanisms in muscle disease. 

## Supplementary Information

Supplementary Material 1.

## Data Availability

Raw data has been submitted to ‘PRoteomics IDEntifications database’ (http://www.ebi.ac.uk/pride), analysed data can be requested from the corresponding author.
