# Supplementary material for: Spatial protein expression patterns across pathologically-associated fibers revealed molecular specialization in inclusion body myositis
Source: Cell Commun Signal. 2026 Feb 21;24:200. doi: 10.1186/s12964-026-02720-7 (PMC13032386; doi:10.1186/s12964-026-02720-7)
Supplement: Supplementary file 1 — Supplementary Material 1. [file 12964_2026_2720_MOESM1_ESM.docx]

**Supplementary Tables, Figures and Methods**

**Table S1: Patient information**

|  | **Patient 1** | **Patient 2** | **Patient 3** |
| --- | --- | --- | --- |
| **Gender** | M | F | M |
| **Age** | 60 | 63 | 58 |
| **History** | Diabetes, Hypertension | Unremarkable | Unremarkable |
| **Months since complaints** | 22 | 48 | 24 |
| **MRI** | Oedema in upper leg, fatty infiltration mainly in lower leg | Patchy Oedema and fatty infiltration | Oedema and fatty infiltration in upper leg muscle |
| **MRC*** | 4+ | 4- | 4+ |

* A neurological assessment of muscle strength , scale 1-5

**Table S2: Antibodies**

|  | **Host** | **Dilution** | **Source (Company, Cat nr)** |
| --- | --- | --- | --- |
| **Primary antibodies** | | | |
| MYH3 | Mouse | 1:20 | DSHB, BF-45 |
| Laminin | Rabbit | 1:2000 | Abcam, ab30320 |
| Dystrophin | Mouse | 1:500 | DSHB, 7A10 |
| Collagen | Goat | 1:50 | Southern Biotech, 1301-01 |
| HNRNPA1 | Rabbit | 1:1000 | CUSABio, CSB-PA010600HA01HU |
| **Secondary antibodies** | | | |
| Anti-Goat-Alexa488 | Donkey | 1:2000 | ThermoFisher Scientific, A32814 |
| Anti-Mouse-Alexa488 | Goat | 1:1000 | ThermoFisher Scientific, A32723 |
| Anti-Mouse-Cy5 | Goat | 1:1000 | Southern Biotech, 6410-15 |
| Anti-Rabbit-Cy5 | Goat | 1:1000 | ThermoFisher Scientific, A10523 |
| Anti-Rabbit-Cy7 | Donkey | 1:1000 | ThermoFisher Scientific, A21039 |
| **Homemade conjugated antibodies** (from [1] | | | |
| MyHC1-Alexa 350 | Mouse | 1:100 | DSHB, BA-D5 |
| MyHC2a-Alexa 594 | Mouse | 1:700 | DSHB, SC-71 |

**Table S3: Significantly enriched GO pathways between control and IBM**

|  |  | **gene**  **count** | | **gene set**  **size** | **strength** | **signal** | **FDR** |
| --- | --- | --- | --- | --- | --- | --- | --- |
| **Downregulated** | |  | |  |  |  |  |
| GO:0043292 | Contractile fiber | 37 | | 246 | 1.05 | 3.21 | 1.36E-23 |
| GO:0030016 | Myofibril | 36 | | 237 | 1.05 | 3.19 | 4.26E-23 |
| GO:0030017 | Sarcomere | 34 | | 217 | 1.07 | 3.17 | 3.68E-22 |
| GO:0015629 | Actin cytoskeleton | 38 | | 482 | 0.77 | 1.87 | 1.22E-15 |
| GO:0098800 | Inner mitochondrial membrane protein complex | 19 | | 158 | 0.95 | 1.77 | 2.35E-10 |
| GO:0016460 | Myosin II complex | 10 | | 26 | 1.46 | 2.12 | 1.97E-09 |
| GO:0031674 | I band | 17 | | 146 | 0.94 | 1.59 | 4.04E-09 |
| GO:0030018 | Z disc | 15 | | 131 | 0.93 | 1.43 | 6.25E-08 |
| GO:0016459 | Myosin complex | 11 | | 56 | 1.17 | 1.61 | 7.07E-08 |
| GO:0005746 | Mitochondrial respirasome | 13 | | 94 | 1.01 | 1.47 | 9.88E-08 |
| GO:0005743 | Mitochondrial inner membrane | 27 | | 502 | 0.6 | 1.05 | 1.29E-07 |
| GO:0031966 | Mitochondrial membrane | 33 | | 752 | 0.52 | 0.93 | 2.46E-07 |
| GO:0098803 | Respiratory chain complex | 12 | | 90 | 1 | 1.34 | 5.24E-07 |
| GO:0062023 | Collagen-containing extracellular matrix | 23 | | 407 | 0.62 | 1 | 7.48E-07 |
| GO:0031012 | Extracellular matrix | 25 | | 552 | 0.53 | 0.82 | 7.73E-06 |
| GO:0005759 | Mitochondrial matrix | 16 | | 494 | 0.38 | 0.35 | 2.17E-02 |
| **Upregulated** | |  |  | |  |  |  |
| GO:1990904 | Ribonucleoprotein complex | 19 | 687 | | 0.65 | 0.89 | 8.02E-06 |
| GO:0005730 | Nucleolus | 17 | 996 | | 0.44 | 0.42 | 8.30E-03 |
| GO:0140535 | Intracellular protein-containing complex | 13 | 784 | | 0.43 | 0.3 | 4.53E-02 |

**Table S4: Overlapping proteins from literature study**

Sarcomeric proteins are highlighted in yellow.

Proteins that were found as myofiber-specific are highlighted: eMyH-F in green .

| **Upregulated** | | **Downregulated** | | |  |
| --- | --- | --- | --- | --- | --- |
| Nijssen & Guttsches | Nijssen, Parker & Guttsches | Nijssen & Parker | Nijssen & Li | Nijssen, Parker & Li | Neijssen & de Vries |
| CKB | VIM | ACTN3 | MYH2 | TNNT3 | MYL6 |
| EEF1B2 |  | MYBPC2 | MYH7 |  | CLU |
| HNRNPK |  | MYH1 | TNNI1 |  |  |
| MGST2 |  | MYOM1 | FGB |  |  |
| RPL23A |  | MYOM2 |  |  |  |
| RPS6 |  | MYOM3 |  |  |  |
| STIP1 |  | MYOZ1 |  |  |  |
| TAGLN2 |  | NEB |  |  |  |
| USP5 |  | TNNC2 |  |  |  |
|  |  | TNNI2 |  |  |  |
|  |  | TNNT1 |  |  |  |
|  |  | TRIM72 |  |  |  |
|  |  | TTN |  |  |  |
|  |  | AGL |  |  |  |
|  |  | ATP2A1 |  |  |  |
|  |  | LDB3 |  |  |  |
|  |  | PYGM |  |  |  |

**Table S5: Unique protein signatures among differentially expressed proteins**

| **Unique to eMyH-F** | | | | **Unique to CN-F** | | **Unique to Inf-F** | |
| --- | --- | --- | --- | --- | --- | --- | --- |
| ACAA1 | ESD | MYDGF | SF3B4 | ACO2 | RPL7 | ACTN1 | OTUB1 |
| ACAT2 | FARSA | **MYH3** | SH3BGRL | APOH | RPS18 | ARHGDIB | PAICS |
| ACO1 | GAA | **MYH8** | SMG8 | ARHGDIA | SLC25A6 | ARHGEF26 | POF1B |
| ACOX1 | GBE1 | MYL12A | SMPD1 | ASTN2 | SOD2 | ASPRV1 | RAB10 |
| AHSG | GCN1 | MYL6B | SNRPB | ATP5F1C | SORD | ATR | RAB5B |
| ANXA6 | GLA | MYO18A | SNRPD2 | C2CD5 | SPATA5L1 | CBR1 | RAC1 |
| APMAP | GMPR2 | NAGA | SPTAN1 | CMYA5 | SPRR2F | CCT4 | RACK1 |
| APOE | GNS | NAPA | STS | COX4I1 | SRSF5 | CEACAM5 | RPS11 |
| ARCN1 | GOT2 | NDRG2 | SUB1 | EIF5A | TRAP1 | CLTC | RPS13 |
| ARPC2 | H1-4 | NUCKS1 | SUMF2 | GTF2H1 | TRIM29 | CMPK1 | RPS16 |
| BANF1 | HMGB2 | NUDT16 | SYNGR2 | HNRNPA2B1 | TRIP10 | CNOT3 | RRBP1 |
| CANX | HPRT1 | OBSCN | SYPL1 | HOOK3 | TUBB2A | COL3A1 | SCARB2 |
| CAPNS2 | HPSE | PCBP1 | TAGLN2 | IGHV3-74 | UBA1 | CSMD2 | SLC25A3 |
| CHD2 | HRG | PGLS | THOP1 | IGKV1-5 | XYLT2 | ECH1 | SPRR1A |
| CHI3L1 | HSPB8 | PITPNA | TM7SF2 | MBP | ZNF185 | EIF3A | SPRR1B |
| CHI3L2 | IGHV3-49 | PLA2G4D | TM9SF3 | MORC1 |  | ENO3 | TUBB3 |
| CHMP1B | IGKV3D-20 | PLP2 | TMED3 | MYEF2 |  | EVPL | USP17L1 |
| CORO1A | IGLV2-8 | PPP1CA | TMED7 | MYOM2 |  | FGA | VSIG8 |
| CPNE1 | IMPA1 | PRDX6 | TMED9 | NEFH |  | G6PD | YWHAQ |
| CRYBG1 | KIF5B | PSAP | TMEM106B | NUCB2 |  | GNB1 |  |
| CST1 | KLK9 | PSMB8 | TPP2 | PABPC1 |  | HADHA |  |
| CTNNA1 | KPNB1 | PSMD6 | UBE2V1 | PDLIM5 |  | HADHB |  |
| CYP2U1 | LGALSL | RAB21 | UFC1 | PPA1 |  | HNRNPA1 |  |
| DCTN1 | LRBA | RASAL1 | UFD1 | PRB2 |  | HSP90AB1 |  |
| DPP7 | LXN | RCC2 | UQCRC1 | PRB3 |  | IDH2 |  |
| DSTN | LYPLA1 | RNASE4 | UQCRH | PSMD11 |  | JUP |  |
| DYNC1H1 | MAN2B2 | RNH1 | USP15 | PSMD14 |  | KATNAL2 |  |
| ECE2 | MGST2 | RPIA | VIM | PYGM |  | KLHL41 |  |
| EMC1 | MMP9 | RUVBL1 | VPS26A | RAD50 |  | MPO |  |
| EML2 | MPST | S100A7A | VWA5A | RAP1A |  | MRPS27 |  |
| EPS8L1 | MTAP | SELENBP1 | YBX3 | RNASE1 |  | MUC5AC |  |

**Table S6: Enriched GO pathway in signatures of pathology-associated myofibers**

A list of the significantly enriched GO-terms using *STRING* analysis.

**eMyH-F**

| GO-term | Description | gene count | background | strength | signal | FDR |
| --- | --- | --- | --- | --- | --- | --- |
| GO:0034774 | Secretory granule lumen | 33 | 321 | 0.89 | 2.24 | 9.46e-17 |
| GO:0005764 | Lysosome | 41 | 746 | 0.62 | 1.38 | 2.44e-12 |
| GO:0042582 | Azurophil granule | 20 | 154 | 0.99 | 1.99 | 1.08e-11 |
| GO:0005775 | Vacuolar lumen | 19 | 175 | 0.92 | 1.66 | 7.39e-10 |
| GO:0101002 | ficolin-1-rich granule | 19 | 185 | 0.89 | 1.58 | 1.73e-09 |
| GO:0035578 | Azurophil granule lumen | 13 | 91 | 1.04 | 1.52 | 6.24e-08 |
| GO:1904813 | ficolin-1-rich granule lumen | 14 | 124 | 0.93 | 1.35 | 2.04e-07 |
| GO:0000502 | Proteasome complex | 10 | 61 | 1.1 | 1.33 | 1.34e-06 |
| GO:0043202 | Lysosomal lumen | 10 | 97 | 0.89 | 0.94 | 5.66e-05 |

**CN-F**

| GO-term | Description | gene count | background | strength | signal | FDR |
| --- | --- | --- | --- | --- | --- | --- |
| GO:0034774 | Secretory granule lumen | 24 | 321 | 0.89 | 1.83 | 4.65e-12 |
| GO:0101002 | ficolin-1-rich granule | 16 | 185 | 0.96 | 1.56 | 8.77e-09 |
| GO:0042582 | Azurophil granule | 14 | 154 | 0.98 | 1.46 | 6.29e-08 |
| GO:1904813 | ficolin-1-rich granule lumen | 12 | 124 | 1.01 | 1.35 | 4.79e-07 |
| GO:0005764 | Lysosome | 27 | 746 | 0.58 | 0.97 | 3.62e-07 |
| GO:0005775 | Vacuolar lumen | 11 | 175 | 0.82 | 0.87 | 9.05e-05 |
| GO:0030016 | Myofibril | 12 | 237 | 0.72 | 0.76 | 0.00023 |

**Inf-F**

| GO-term | Description | gene count | background | strength | signal | FDR |
| --- | --- | --- | --- | --- | --- | --- |
| GO:0005840 | Ribosome | 11 | 228 | 0.9 | 0.99 | 2.59e-05 |
| GO:1990904 | Ribonucleoprotein complex | 18 | 687 | 0.63 | 0.82 | 2.63e-05 |
| GO:0044391 | Ribosomal subunit | 10 | 187 | 0.94 | 1.0 | 3.28e-05 |
| GO:0034774 | Secretory granule lumen | 12 | 321 | 0.79 | 0.86 | 7.37e-05 |

**Table S7: Aggregation-prone protein list**

| **eMYH-F** | | **CN-F** | | **Inf-F** | |
| --- | --- | --- | --- | --- | --- |
| ACADVL | PSMC6 | ACO2 | PSMC6 | ACADVL | RPS16 |
| ARCN1 | PSMD6 | ANXA11 | PSMD11 | ANXA11 | RPS3 |
| ARPC2 | RAD23B | CCT8 | PSMD14 | CCT4 | RRBP1 |
| CCT8 | RAN | CRNN | PYGM | EIF2S1 | SLC25A3 |
| CRNN | RPN1 | FAM184A | RAD23B | EIF3A | TXNL1 |
| EIF2S1 | RPS3 | HNRNPA2B1 | RAN | EVPL |  |
| FAM184A | RPS5 | HNRNPH1 | RPL31 | HADHA |  |
| GCN1 | RPSA | HNRNPU | RPL7 | HADHB |  |
| GMPR2 | RPTN | HSPA9 | RPN1 | HNRNPA1 |  |
| HNRNPH1 | RUVBL1 | HSPD1 | RPS5 | HNRNPU |  |
| HSPA9 | SDHA | IVL | RPSA | IVL |  |
| HSPD1 | SPRR4 | MSN | RPTN | LAMB2 |  |
| KIF5B | SPTAN1 | PDCD6IP | SDHA | PHB2 |  |
| LAMB2 | SPTBN1 | PHB2 | SPRR4 | RACK1 |  |
| MSN | TXNL1 | PRB2 | SPTBN1 | RPL31 |  |
| PDCD6IP |  | PRB3 | UBA1 | RPS11 |  |
| PSMC3 |  | PSMC3 |  | RPS13 |  |

**Supplementary Figures:**

**Figure S1. A Flowchart summary of the wet lab procedures and analysis steps in myofiber-level analysis.**

**
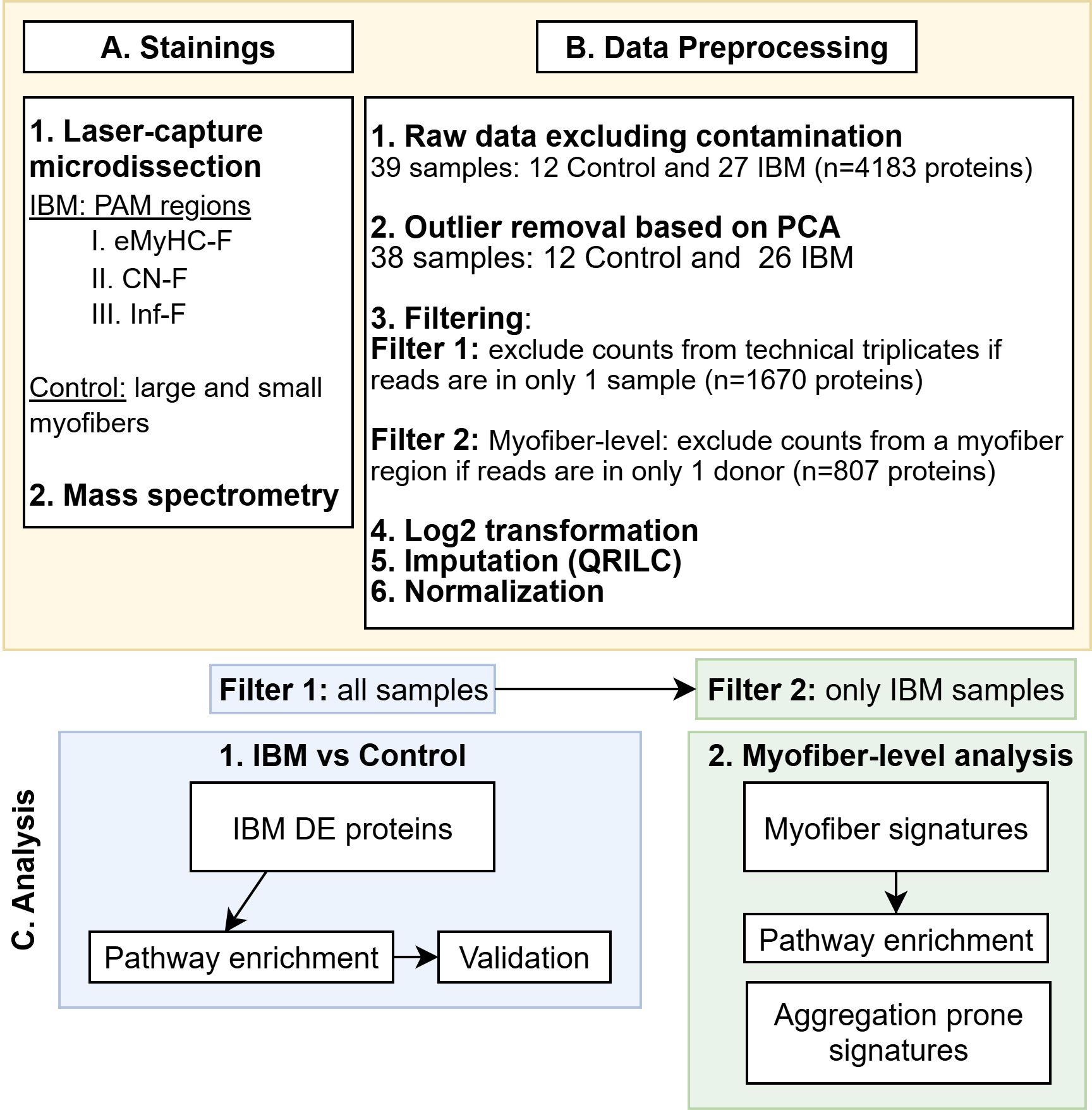
** Staining and preprocessing are depicted in yellow. Data analyses: 1. IBM *vs* Control in blue, 2. myofiber regions in yellow, and protein aggregation signatures in purple.


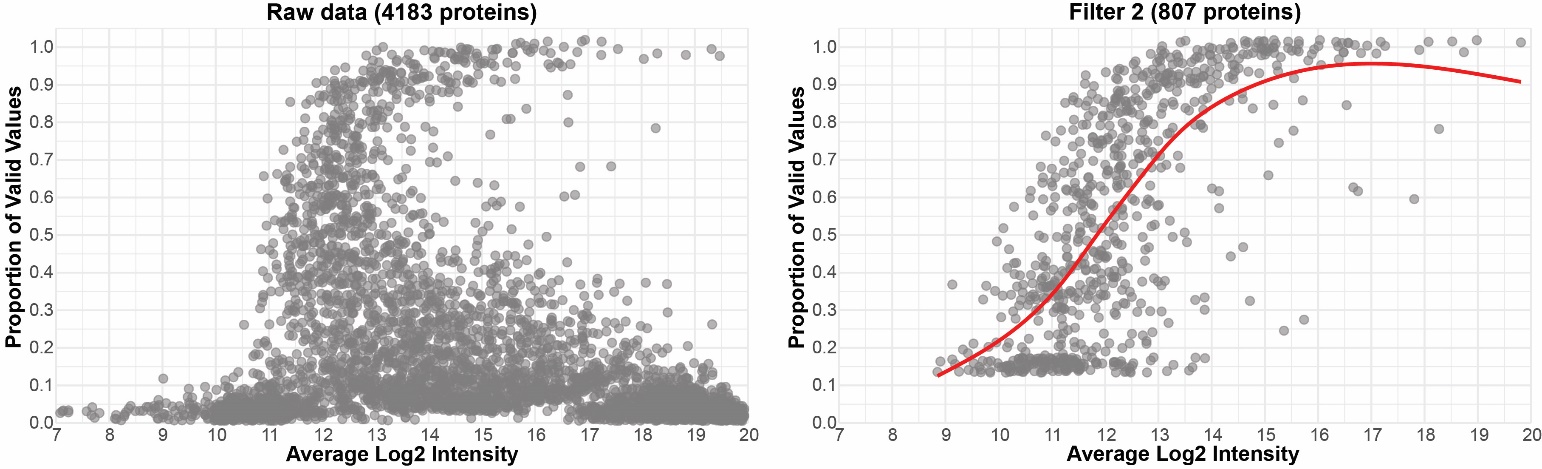
**Figure S2: The fitness of dataset distribution to the imputation model.**
A scatterplot of the proportion of valid values for raw data and after filter-2. Jittering is added to reduce over-plotting. A red line shows the cubic spline fitted to data distribution for filter-2, showing that missing data is intensity-dependent.


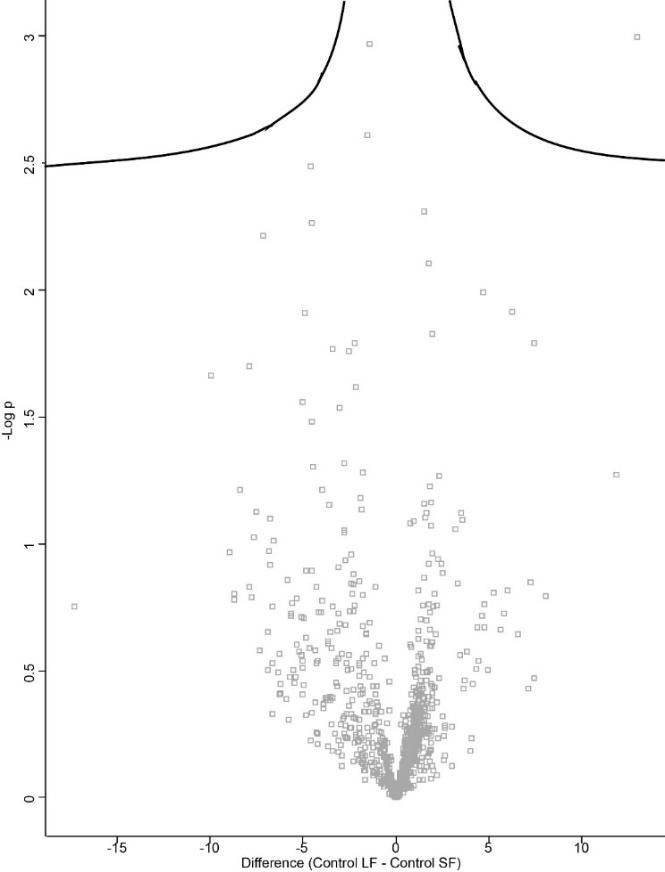


**Figure S3. Volcano plot of DEA between small and large myofibers in control samples**. The black line depicts p<0.05, FDR.


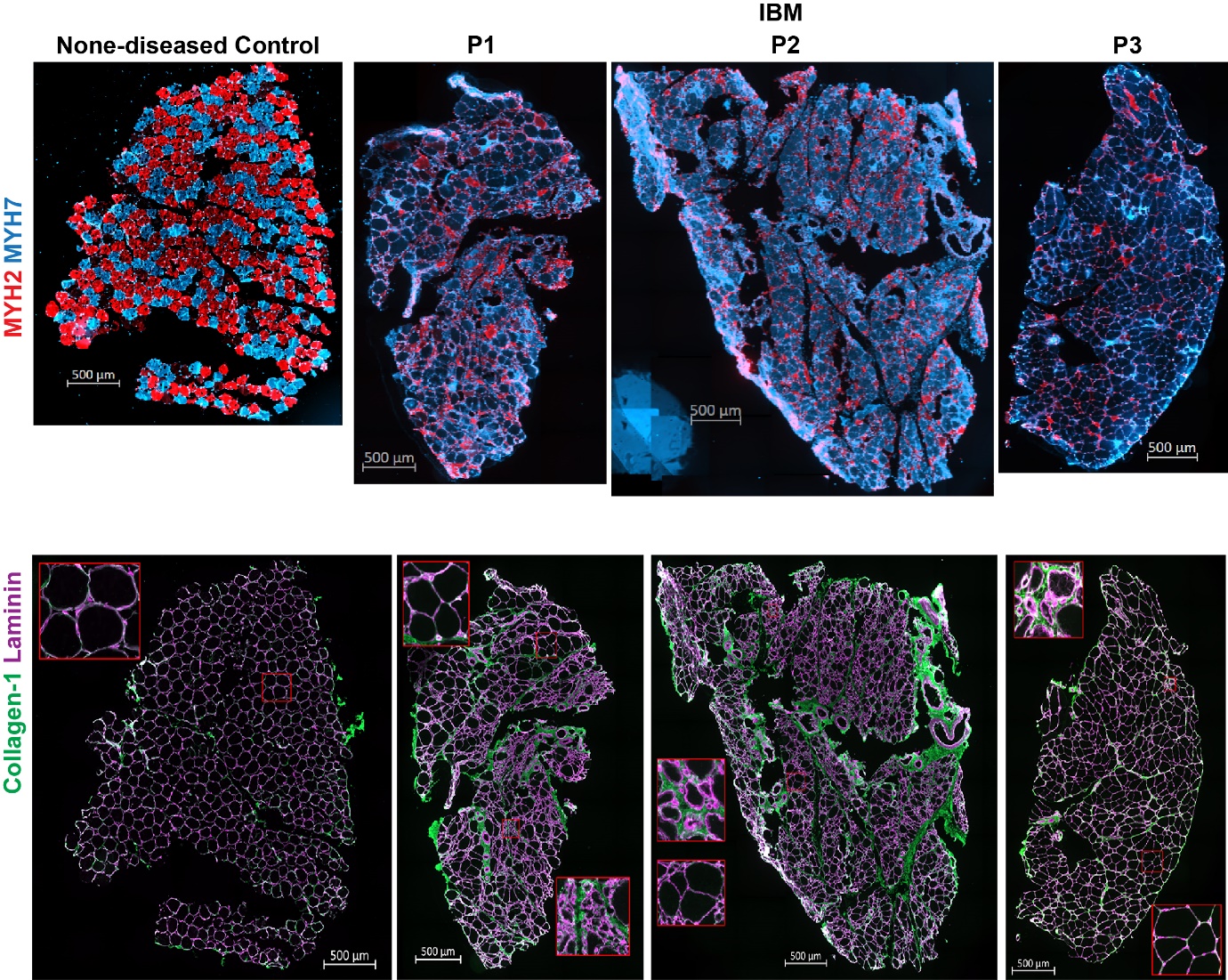


**Figure S4. Images of immunofluorescence of the entire cross-section.**

Upper row: immunofluorescence with MHY2 and MHY7; lower row immunofluorescence with Collagen-1 and laminin in control and IBM muscles. P2 showed the most severe muscle histopathology. Insert shows magnification of the selection region (insert in red). Scale bar: 500µm.


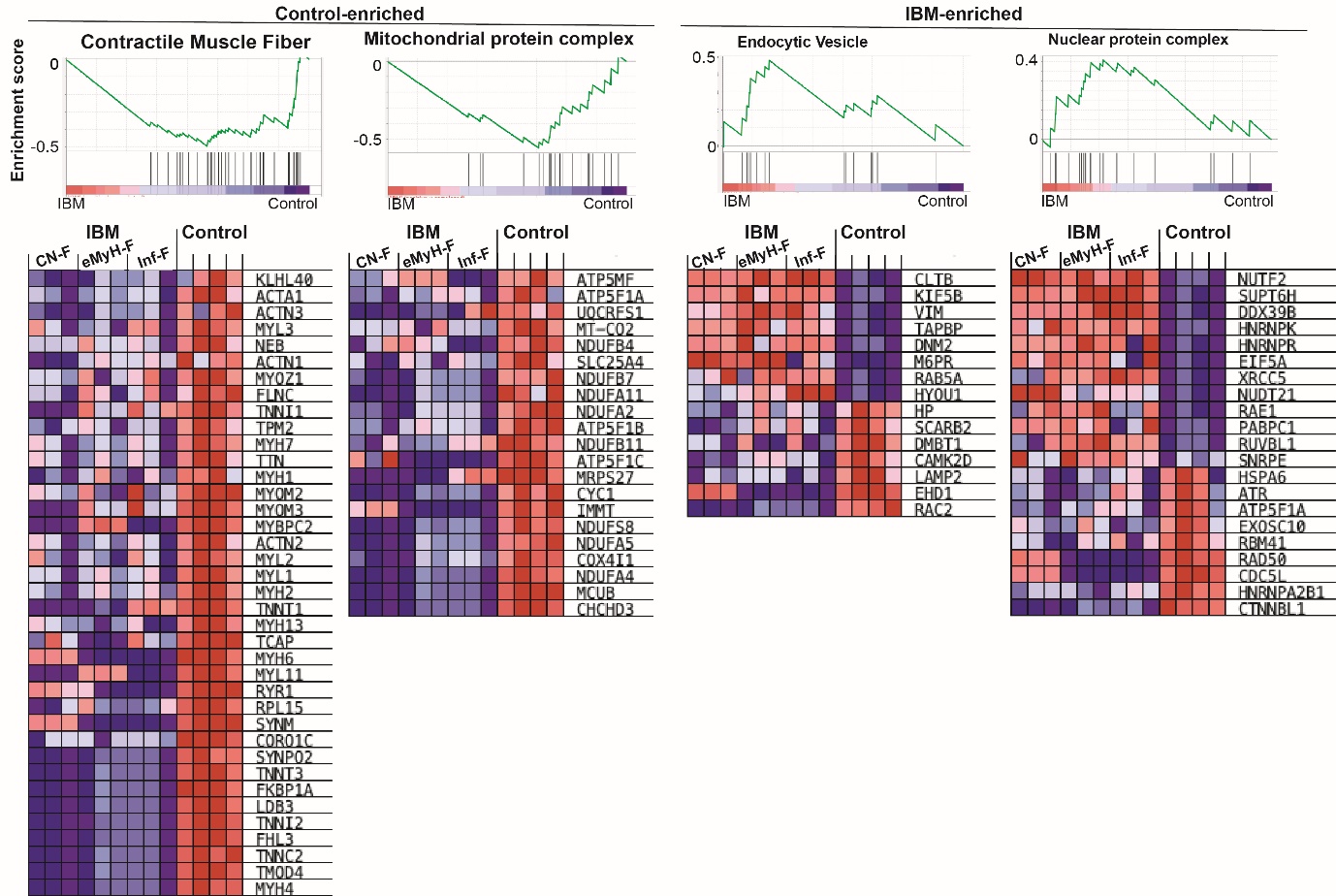


**Figure S5. Heatmaps of the significantly enriched gene networks between control and IBM.** GSEA heatmaps of the gene networks (GO:Cellular Component) in Figure 2D. The proteins positively correlated with IBM are depicted in red and the negatively correlated in blue.

**
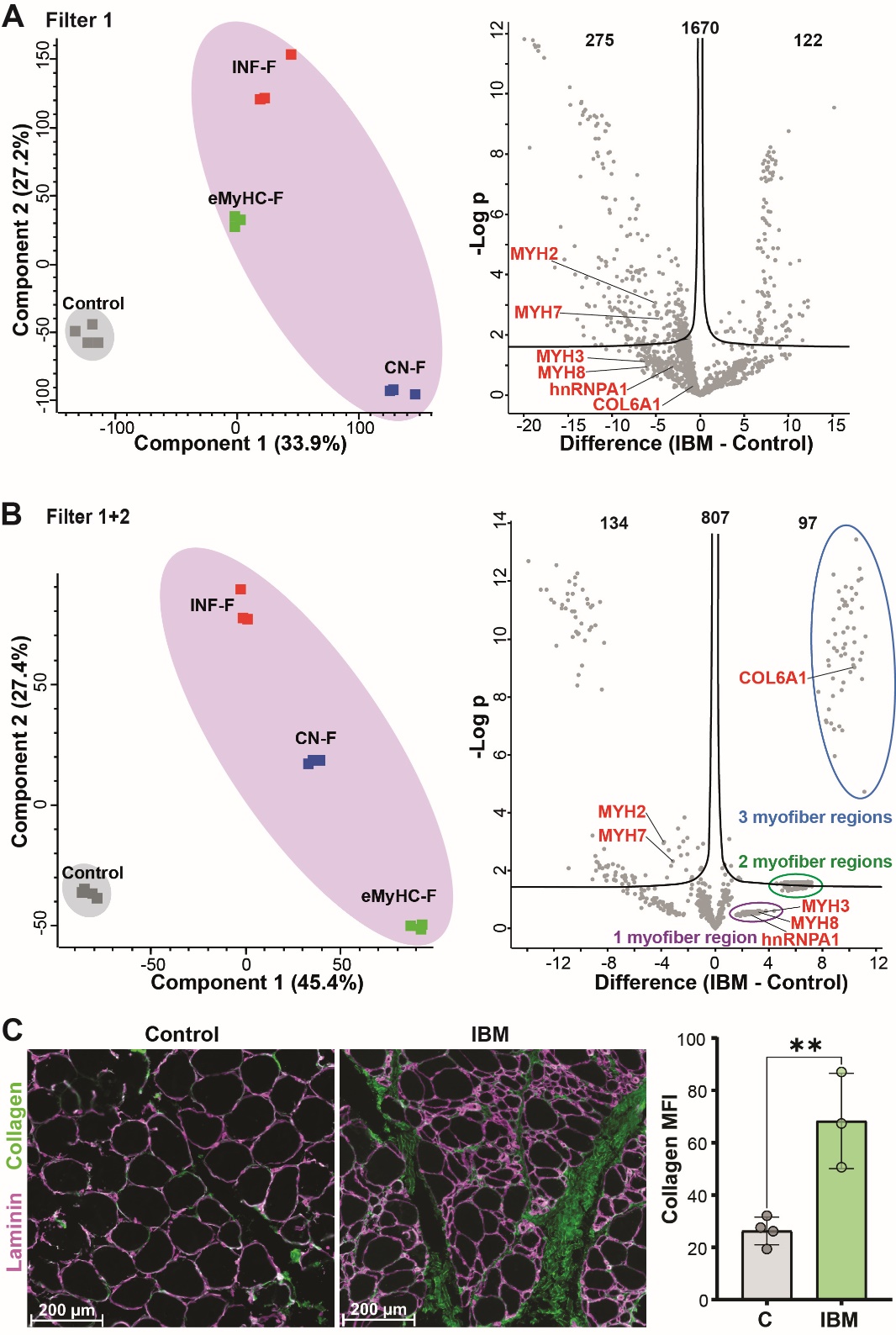
**

**Figure S6. PCA and Volcano plots in filter-1 and filter-2 datasets.**
**A.** Principal component analysis and volcano plot of dataset after filter 1. PCA shows variation between myofiber subtypes in control and IBM samples. Control samples are encircled in grey; IBM are encircled in pink. Volcano plot shows differentially expressed proteins; black line shows p-value of 0.05 with FDR correction. **B.** Principal component analysis and volcano plot of dataset after filter 1 and 2. PCA shows variation between myofiber subtypes in control and IBM samples. Control samples are encircled in grey; IBM are encircled in pink. Volcano plot shows differentially expressed proteins; black line shows p-value of 0.05 with FDR correction. Annotated are the clouds in the volcano plot that show protein only found in one (purple), two (green) or all three (blue) of the selected myofiber regions. **C.** Representative images from control and IBM muscle cryosection immunostained for laminin (pink) and collagen (green). Scale bar: 200μm. Bar chart shows collagen mean florescence intensity (MFI) in control (C, N=4) and IBM (N=3). Statistical significance was assessed with one-way Anova, ** depicts p<0.01


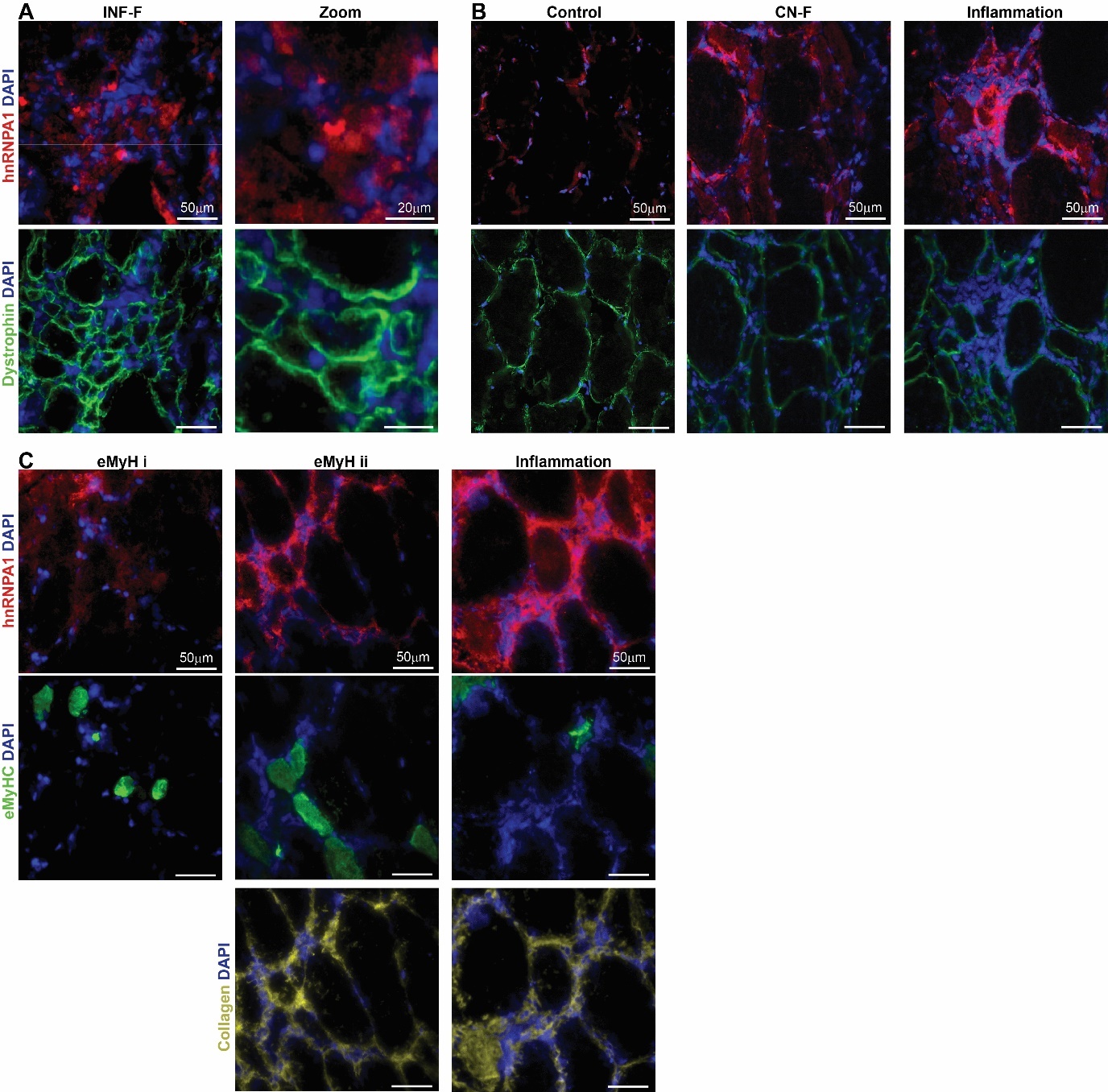


**Figure S7. Details of HNRNPA1 immunofluorescence in IBM muscles.** Details of figure 5 showing an overlay of one antibody and DAPI. Scale bar: 50μm.


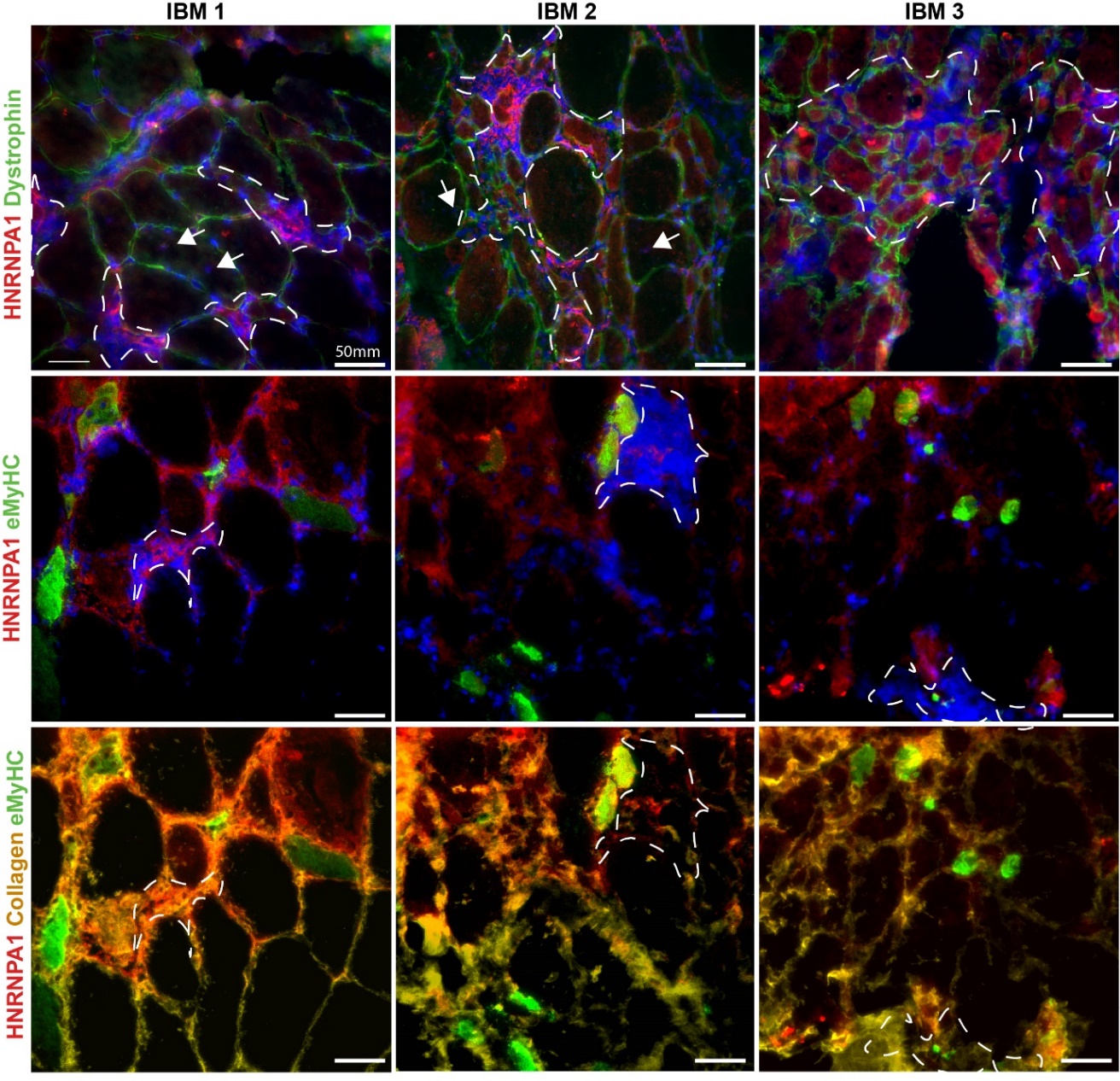


**Figure S8. HNRNPA1 immunofluorescence in IBM muscles.** Representative images from three different IBM patients. The upper row shows immunofluorescence with dystrophin (green), and the middle row shows immunofluorescence with eMyHC (green). The lower row shows immunofluorescence with eMyHC (green) and Collagen (yellow). HNRNPA1 is shown in red, and the nuclei in blue. A region of inflammation is marked with a dashed line. White arrows indicate central nuclei, and yellow arrows indicate eMyHC myofibers. Scale bar: 50μm.


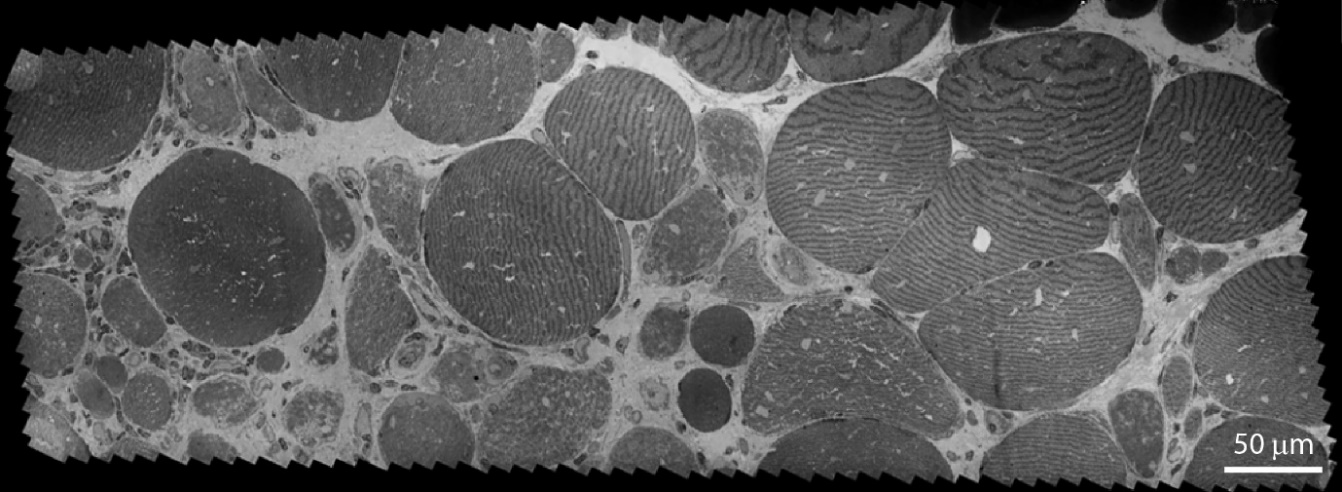


**Figure S9. An overview of IBM VL muscle.** A stitched electron microscope. Scale bar: 50µm.

1 Riaz M, Raz Y, van Putten M, et al. PABPN1-Dependent mRNA Processing Induces Muscle Wasting. PLoS Genet 2016;12(5):e1006031.
